# An inner membrane complex protein IMC1g in *Plasmodium berghei* is involved in asexual stage schizogony and parasite transmission

**DOI:** 10.1128/mbio.02652-24

**Published:** 2024-11-22

**Authors:** Yinjie Liu, Shitong Cheng, Gang He, Dawei He, Duo Wang, Sicong Wang, Lumeng Chen, Liying Zhu, Yonghui Feng, Liwang Cui, Yaming Cao, Xiaotong Zhu

**Affiliations:** 1 Department of Immunology, College of Basic Medical Sciences, China Medical University, Shenyang, China; 2 Department of Laboratory Medicine, the First Hospital of China Medical University, Shenyang, Liaoning, China; 3 Department of Internal Medicine, Morsani College of Medicine, University of South Florida, Tampa, Florida, USA; University of Geneva, Geneva, Switzerland

**Keywords:** IMC1, inner membrane complex, malaria, gliding motility, * Plasmodium berghei*

## Abstract

The inner membrane complex (IMC), a double-membrane organelle underneath the plasma membrane in apicomplexan parasites, plays a significant role in motility and invasion and confers shape to the cell. We characterized the function of PbIMC1g, a component of the IMC1 family member in *Plasmodium berghei*. PbIMC1g is recruited to the IMC in late schizonts, activated gametocytes, and ookinetes. Pairwise yeast two-hybrid assays demonstrate that PbIMC1g interacts with IMC1c, a component of the PHIL1 complex, and the core sub-repeat motif “EKI(V)V(I)EVP” in PbIMC1g is essential for this interaction. Localization of PbIMC1g to the IMC was dependent on its IMCp domain, while its C-terminus and palmitoylation sites were required for the full efficiency of proper IMC targeting. PbIMC1g is required for asexual stage development, and its conditional knockdown resulted in a defect in schizogony. Additionally, PbIMC1g was also important for male gametogenesis and ookinete development. As an IMC component that assists in anchoring the glideosome to the subpellicular network, PbIMC1g was also involved in ookinete motility and mosquito midgut invasion. IMC1g from the human parasite *Plasmodium vivax* could functionally replace PbIMC1g in *P. berghei*, confirming the evolutionary conservation of IMC1g proteins in *Plasmodium* spp. Together, this work reveals an essential role of IMC1g in the parasite life cycle and suggests that IMC1 family members likely contribute to parasite gliding and invasion.

**IMPORTANCE:**

The malaria parasite’s inner membrane complex is critical to maintain its structural integrity and motility. Here, we identified the function of the IMC1g protein, a member of the IMC1 family, in invasive and proliferative stages of *P. berghei*. We found that the IMCp domain of PbIMC1g is critical for proper IMC targeting, and PbIMC1g interacts with PbIMC1c. Conditional knockdown of PbIMC1g expression affects schizogony, gametogenesis, and ookinete conversion. PbIMC1g interacts with IMC1c to firmly anchor the glideosome to the subpellicular network. Additionally, we confirmed that IMC1g is functionally conserved in *Plasmodium* spp. These data reveal the function of IMC1g protein in anchoring the glideosome, providing further insight into the mechanism of the glideosome function.

## INTRODUCTION

*Plasmodium* spp., the causative agent of malaria, caused approximately 246 million cases and 608,000 deaths in 2022 (1). The parasite requires a human host and mosquito vector to complete its complex life cycle. In the human blood, the parasite undergoes many rounds of asexual replication, which causes clinical symptoms of malaria (2). A subset of the parasite differentiates into gametocytes, the sexual form required for transmission to mosquitoes. Once ingested by an anopheline mosquito, gametocytes in the blood bolus immediately transform into gametes, which are fertilized to form zygotes. Within 24 h, the spherical zygotes differentiate into crescent-shaped motile ookinetes, which invade the mosquito midgut epithelium (3, 4). Subsequent sporogonic development results in thousands of sporozoites, which later invade the salivary glands, ready to infect the human host (5, 6). Malaria parasites have several invasive zoite forms during their life cycle, requiring motility to invade host cells. Thus, understanding the mechanism of motility may facilitate the discovery of novel anti-malarial targets.

The gliding motility of invasive stages of the malaria parasite is powered by an actin-myosin motor, termed the glideosome, which is comprised of class XIV myosin A (MyoA), MyoA tail domain-interacting protein (MTIP), essential light chain, and three gliding-associated proteins (GAPs) GAP45, GAP50, and GAP40. The glideosome is anchored to the inner membrane complex (IMC) situated directly underneath the parasite plasma membrane (7, 8). On the cytosolic side of the IMC, it is associated tightly with the subpellicular protein network (SPN), which supports the IMC and provides tensile strength to the parasite (9). Besides the critical role of IMC in regulating gliding motility (10, 11), it also confers stability to cellular architecture (12) and provides a scaffolding framework in parasite division (13–15). The photosensitized INA-labeled protein 1 (PHIL1) complex, formed by IMC1c, PhIL1, PhIL1-interacting protein (PhIP), and glideosome-associated proteins (GAPMs) 1, 2, and 3, is a newly identified glideosome-associated protein complex located in the IMC (15–18). Genetic disruption of PhIL1 prevents the formation of transmittable gametocytes (17). Conditional KD of IMC1c completely blocks asexual stage proliferation, and PhIP KD causes significant defects in schizont segmentation (16, 19). The GAPMs constitute a three-member family of six transmembrane proteins in Apicomplexa (10). Functional studies showed that GAPM1 depletion resulted in the depolymerization of microtubules, compromising the parasite’s shape and integrity (20), whereas merozoites lacking GAPM2 could not invade red blood cells (RBCs) (16). Co-immunoprecipitation (co-IP) studies showed that GAPMs interact with the alveolins on SPN and the glideosome components in the IMC (10). However, how GAPMs regulate the parasite gliding motility is unknown.

Alveolins (or IMC1 proteins) are major SPN components, broadly conserved throughout the alveolate and important for IMC-SPN interconnection (9, 21–23). In *Plasmodium* spp., 13 conserved IMC1 family members, namely, IMC1a-m, have been identified (24). In *P. berghei*, PbIMC1a, PbIMC1b, and PbIMC1h are involved in the gliding motility of sporozoites and ookinetes (25–27). Furthermore, PbIMC1h impacts the directional motility behavior of both these zoite stages (28, 29). *In vitro* assays revealed that *Plasmodium falciparum* IMC1l (PfIMC1l) interacts with actin and MyoA and may directly regulate parasite motility (30). PfIMC1g, which shares 64.1% sequence similarity with PbIMC1g, is not essential for schizogony but may be needed to maintain the structural integrity of merozoites, protecting the parasite from damage during merozoite internalization, since its deficiency results in parasite death shortly after merozoite entry into RBCs (19).

In this study, we performed detailed functional characterization of PbIMC1g in *P. berghei*. PbIMC1g is a palmitoylated protein and is expressed in asexual and sexual development. We show here that PbIMC1g exhibits a typical IMC-like location at the peripheries of merozoites and ookinetes and interacts with the PHIL1 complex component, IMC1c. Using a conditional KD strategy, we show that PbIMC1g plays an important role during the schizogony of the asexual stage, gametogenesis, and ookinete differentiation. In addition, PbIMC1g regulates ookinete motility, possibly through stabilizing the PHIL1 complex in the IMC.

## RESULTS

### IMC1g associates with key IMC components

PbIMC1g (PbANKA_1240600) is predicted to encode a ~34.3 kDa protein lacking transmembrane domains or a signal peptide. IMC1g orthologs are found across *Plasmodium* and other parasitic Apicomplexa, such as *Toxoplasma*, *Babesia*, and *Eimeria* (Fig. S1A). Sequence alignment revealed that PbIMC1g was >88% similar to the orthologs in *Plasmodium yoelii* and *Plasmodium chabaudi* and 63%–69% similar to those in human malaria parasites, like *P. vivax*, *P. falciparum*, and *Plasmodium knowlesi* (Fig. S1B). IMC1g orthologs in *Plasmodium* spp. share a conserved IMCp domain (Pfam 12314) and harbors a core sub-repeat motif “EKI(V)V(I)EVP” within the IMCp domain that defines the alveolins (Fig. S1C).

**Fig 1:**
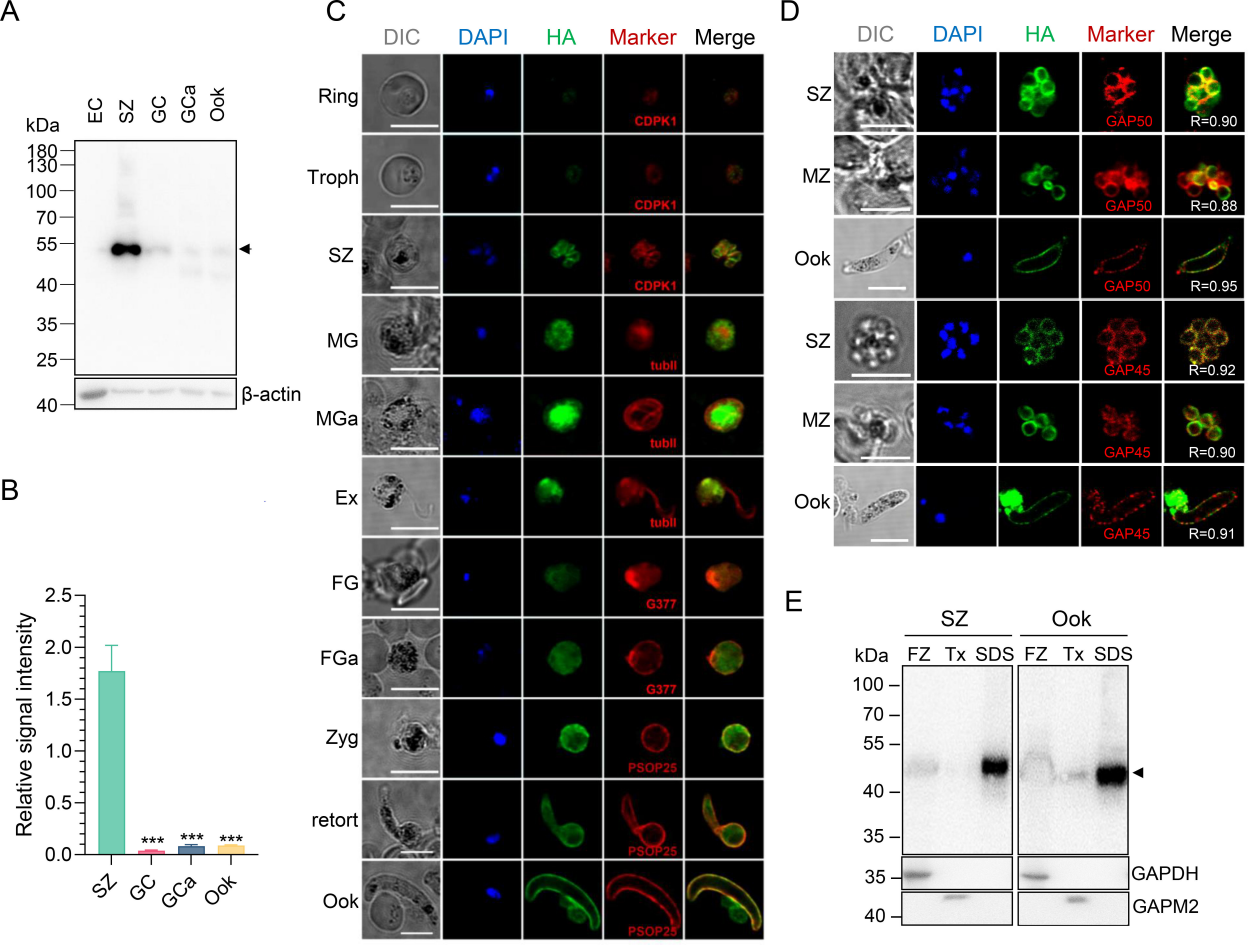
Expression and localization of PbIMC1g. (**A**) Expression of the PbIMC1g-HA protein in schizonts (SZ), gametocytes (GC), activated gametocytes (GCa), and ookinetes (Ook). The arrow indicates PbIMC1g-HA protein (~37.8 kDa) detected using anti-HA mAb. Proteins extracted from uninfected RBCs (EC) were used as a negative control. β-Actin was used as a loading control. (**B**) The relative PbIMC1g-HA/β-actin signal intensity ratios calculated with the Image J software are shown. ***, *P* < 0.001. (**C**) The subcellular localization of PbIMC1g-HA protein during parasite development. Troph, trophozoite; SZ, schizont; MG, male gametocyte; MGa, activated male gametocyte; Ex, exflagellated MG; FG, female gametocyte; FGa, activated female gametocyte; Zyg, zygote; Ook, ookinete. Differential interference contrast (DIC), the DAPI-stained nuclei (blue), PbIMC1g-HA (green), and co-localization markers (red) of the parasites are shown. TubII, α-tubulin II antibody (male-gametocyte/gamete marker); CDPK1, anti-CDPK1 sera (ring/trophozoite stage cytosolic marker); G377, α-Pbg377 sera (female-gametocyte/gamete marker); PSOP25, anti-PSOP25 sera (ookinete surface marker). Scale bar, 5  µm. (**D**) Co-localization of PbIMC1g-HA with glideosome components (GAP45 and GAP50) in schizonts (SZ), merozoites (MZ), and ookinetes (Ook). Nuclei were stained with DAPI. Numbers indicate Pearson’s correlation coefficient values (*R*). Scale bars, 5  µm. (**E**) Western blot analysis of freeze-thaw (FZ), 1% Triton X-100 detergent (Tx), and SDS fractionations shows that PbIMC1g is primarily associated with the cytoskeleton. PbIMC1g-HA band was visualized using mouse anti-HA mAb. GAPDH (cytosolic protein) and GAPM2 (integral membrane protein) were used as FZ and Tx fraction loading controls. The black arrowhead indicates PbIMC1g-HA protein.

To investigate the expression and subcellular location of PbIMC1g, we generated a transgenic parasite line (PbIMC1g^HA^) expressing a triple-HA tag (3×HA) fused with the C-terminus of the endogenous *PbIMC1g* using a marker-free CRISPR-Cas9-based strategy (Fig. S2A). Genomic integration of the tagging construct into the *pbimc1g* locus in *P. berghei* was verified by diagnostic PCR (Fig. S2B). Western blot analysis of PbIMC1g^HA^ parasites with anti-HA tag monoclonal antibody (mAb) confirmed the presence of PbIMC1g-HA protein. However, western blot detected a protein band (PbIMC1g-HA) of ~50 kDa, larger than the expected size of 37.8 kDa, indicating this alveolin migrates slower than its expected mass (Fig. S2B). The HA-fusion protein was detectable from the ring stage onward but drastically increased in the schizont stage (Fig. 1A and B). PbIMC1g was also detectable in gametocytes and ookinetes, although it was far less abundant than in schizonts (Fig. 1A and B). Indirect immunofluorescence assays (IFAs) also detected PbIMC1g as cytosolic punctua in ring and trophozoite stages, while it displayed a cortical localization in merozoites within schizonts, consistent with an SPN localization (Fig. 1C). PbIMC1g-HA was localized in the cytosol of gametocytes, gametes, and zygotes. In exflagellating male gametocytes, it was not prominent on the flagella but associated with the residual body. However, after fertilization, it became predominantly localized to the periphery of zygotes and ookinetes, resembling the localization pattern of the parasite plasma membrane (PPM)-resident protein PSOP25 (31) (Fig. 1C). Furthermore, PbIMC1g exhibited significant co-localization with the IMC markers GAP45 and GAP50 at the periphery of merozoites and ookinetes (Pearson correlation coefficient [PCC] ≥ 0.88), indicating association with the inner membrane complex (Fig. 1D). Next, we wanted to verify the association of PbIMC1g with the IMC. Detergent fractionation assays with PbIMC1g^HA^ parasites at the schizont and ookinete stages showed that PbIMC1 was primarily present in the SDS-soluble fraction as compared to the Triton X-100-soluble fraction, suggesting its association with the cytoskeleton (Fig. 1E).

**Fig 2:**
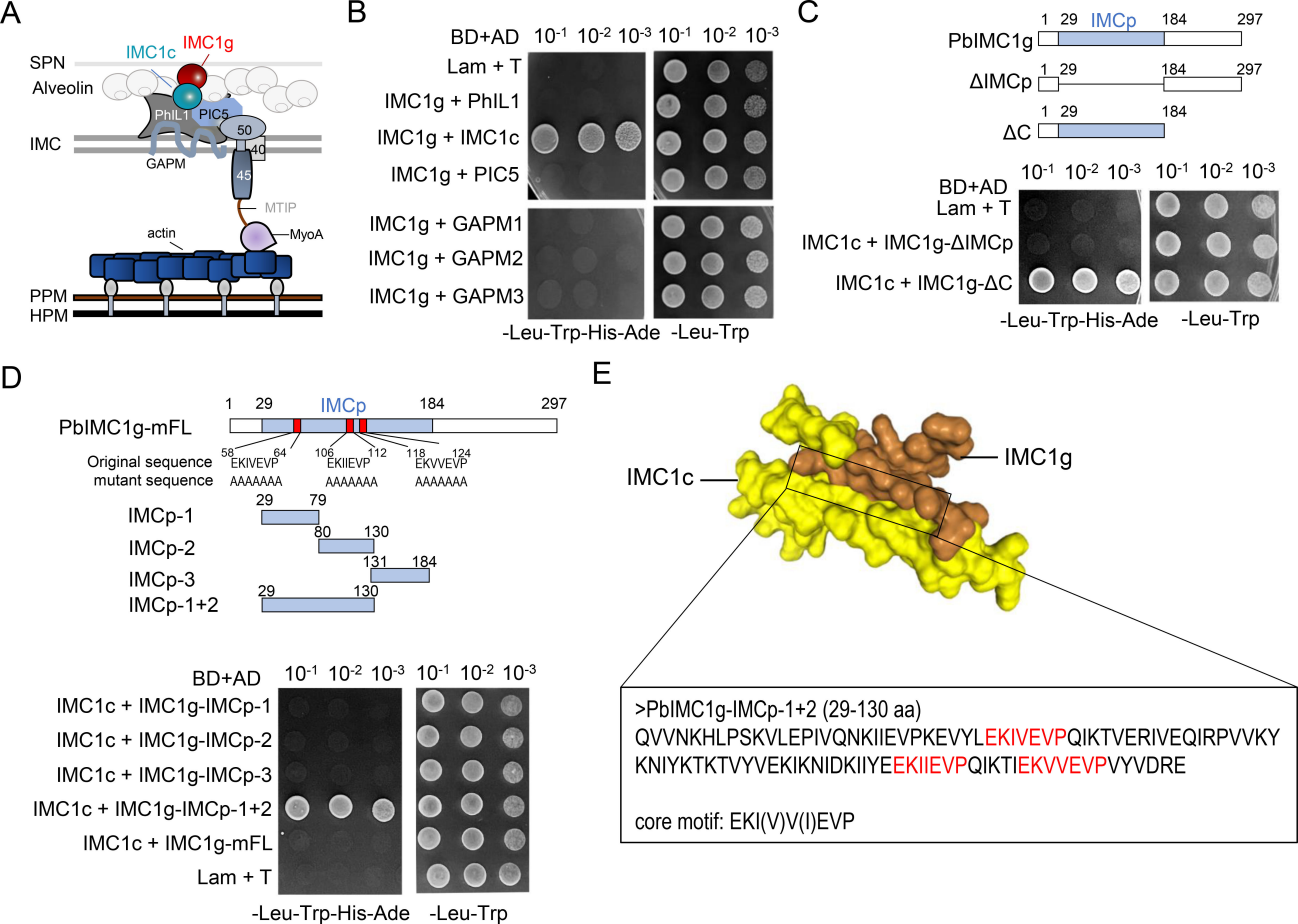
The core sub-repeat motif of PbIMC1g binds to the PbIMC1c protein. (**A**) Schematic model of IMC1g and the main components of PHIL1 complex in IMC. (**B**) Tenfold dilutions of yeast two-hybrid reporter strains containing the indicated bait (BD) and prey (AD) plasmids. Plating onto a synthetic complete medium lacking leucine and tryptophan (–Leu –Trp) selects for the bait and prey plasmids, and plating onto a medium lacking leucine, tryptophan, histidine, and adenine (–Leu –Trp –His –Ade) additionally selects for physical interaction of the bait and prey fusion proteins. Lam, Gal4 BD fused with lamin. T, the SV40 large T antigen. (**C**) Diagram of the ΔIMCp and ΔC truncations of PbIMC1g and PbIMC1c used for pairwise Y2H assays. Residues included in each fragment are indicated. (**D**) Diagram of the four fragments within IMCp and core sub-repeat motif “EKI(V)V(I)EVP” mutated version of PbIMC1g that were individually generated for PbIMC1c binding assay. The core sub-repeat motif within the IMCp domain of PbIMC1g, including the “EKIVEVP” (58–64 aa), “EKIIEVP” (106–112 aa), and “EKVVEVP” (118–124 aa), was mutated to alanine simultaneously, to generate PbIMC1g-mFL construct. IMCp-1, 29–79 aa; IMCp-2, 80–130 aa; IMCp-3, 131–184 aa; IMCp-1+2, 29–130 aa. (**E**) Schematic model of the interaction region between PbIMC1g and PbIMC1c. The core sub-repeat motifs responsible for PbIMC1g and PbIMC1c interaction were marked in red.

*Plasmodium falciparum* IMC1g (PfIMC1g) has previously been reported to interact with IMC1c (16, 19), a component of photosensitized INA-labeled protein 1 (PhIL1)-associated complex (PHIL1) (Fig. 2A). To test the pairwise interaction of PbIMC1g with the PHIL1 complex component, including IMC1c, PhIL1, PIC5, GAPM1, GAPM2, and GAPM3, we performed a yeast two-hybrid (Y2H) assay using PbIMC1g as bait. We cotransformed the six combinations of the PHIL1 components (prey) and PbIMC1g (bait) into yeast and tested each strain’s ability to grow on permissive and restrictive media (Fig. 2B). All six strains grew to thrive on permissive media and were not autoactivating; only the strain expressing PbIMC1g and PbIMC1c grew on restrictive media, indicating that PbIMC1g interacts with IMC1c (Fig. 2B). To identify the regions of PbIMC1g interacting with PbIMC1c, we generated two truncations of PbIMC1g, one removing the IMCp domain (amino acids [aa] 29–184) and another (ΔC) deleting the C-terminal aa 184–297 of PbIMC1g. In Y2H analysis, ΔC still interacted with PbIMC1c, whereas ΔIMCp did not, demonstrating that the IMCp domain is essential for binding to PbIMC1c (Fig. 2C). When four additional IMCp domain truncations of PbIMC1g were tested, only the IMCp-1+2 (aa 29–130) was able to interact with PbIMC1c (Fig. 2D). Additionally, we found that mutating the core sub-repeat motifs, including “EKIVEVP” (aa 58–64), “EKIIEVP” (aa 106–112), and “EKVVEVP” (aa 118–124), within the IMCp-1+2 domain of PbIMC1g completely abolished the interaction between PbIMC1g and PbIMC1c, indicating that the core sub-repeat motif “EKI(V)V(I)EVP” within the IMCp domain is critical for PbIMC1g-PbIMC1c interactions (Fig. 2E).

### The IMCp domain is critical for the SPN association of PbIMC1g

**Fig 3:**
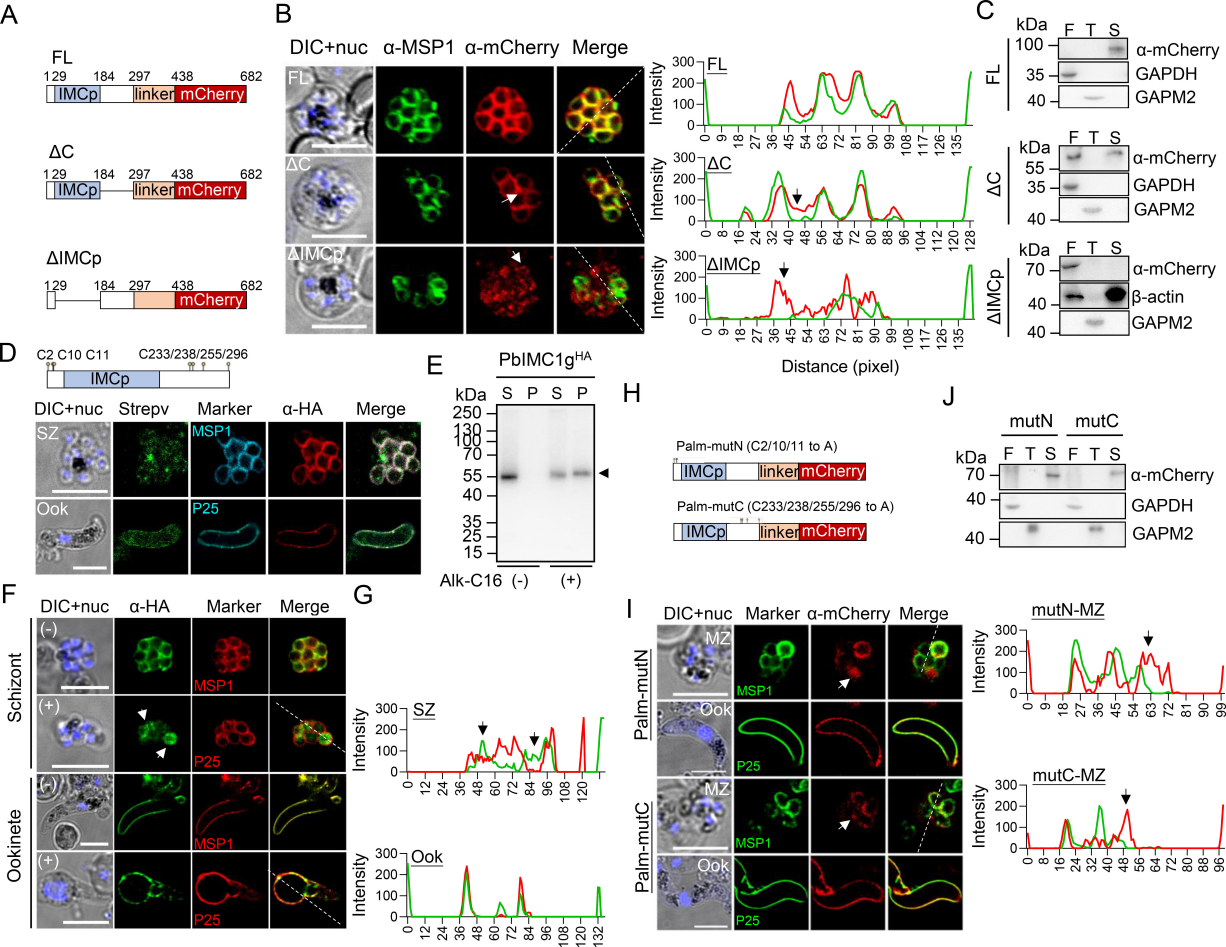
The IMCp domain contributed to the IMC targeting of PbIMC1g. (**A**) Schematic drawing of mcherry-tagged full-length and deletion construct of PbIMC1g protein. (**B**) Fluorescent microscopy of mCherry-tagged PbIMC1g (red) and MSP1 (green) in schizonts of the FL, ΔC, and ΔIMCp parasites. The nucleus (nuc) was stained with DAPI (blue). Scale bar, 5 µm. Plot profiles of signal intensities evaluated by Image J software are shown on the right side of each immunofluorescence panel. The mis-localized signals were marked with arrows. (**C**) Solubility assay detecting membrane association of FL (top), ΔC (middle), and ΔIMCp (bottom) using different detergents. Cytosolic soluble proteins are in freeze-thaw (F), integral membrane proteins in Triton X-100 buffer (T), and peripheral membrane proteins in 2% SDS buffer (S). GAPDH, GAPM2, and β-actin were used as loading controls for F-, T-, and S-fractions, respectively. FL-mcherry, 77.9 kDa; ΔC-mCherry, 65.0 kDa; ΔIMCp-mCherry, 59.5 kDa. (**D**) Click chemistry method detecting palmitoylation of PbIMC1g in PbIMC1g^HA^ parasites in the schizont and ookinete stages. Structure and residues for potential palmitoylated cysteine residues of PbIMC1g were shown in the upper PbIMC1g schematic. Localization of PbIMC1g-HA protein on schizont and ookinetes is shown in the lower panel. The alkynyl palmitic acid (Alk-C16) metabolically labeled parasites were stained with Alexa Fluor 488-conjugated streptavidin (Strepv, green), parasite plasma membrane marker MSP1 (cyan) or P25 (cyan), and anti-HA mAb (α-HA, red). Merged images for Strepv, PPM marker, and α-HA are shown in the right column. Scale bar, 5  µm. (**E**) Immunoblot assay of palmitoylated PbIMC1g proteins in ookinetes using CuAAC-click chemistry. The captured palmitoylated proteins labeled with (+) or without (−) Alk-C16 were analyzed by western blotting using anti-HA mAb. S, IP supernatant; P, IP elution. The arrowhead indicates PbIMC1g-HA protein. (**F**) Indirect immunofluorescence microscopy of the PbIMC1g^HA^ parasites at schizont and ookinete stages after treatment with 100 μM 2‐BP. Scale bars, 5 µm. Arrows indicate mis-localized signals of PbIMC1g-HA protein. (**G**) Plot profiles of signal intensities evaluated by Image J software at merozoite and ookinete stages are shown. The mis-localized signals were marked with arrows. (**H**) Pictorial representations of fusion protein constructs used in the experiments are shown. The mutated palmitoylation sites are labeled in the schematics of the Palm-N and Palm-C proteins, respectively. (**I**) Fluorescent microscopy of mCherry-tagged PbIMC1g (red) and MSP1 (green) or P25 (green) at the merozoite and ookinete stages of the Palm-mutN and Palm-mutC parasites. Nuclei (nuc) were stained with DAPI (blue). Scale bar, 5 µm. Plot profiles of signal intensities evaluated by Image J software are shown on the right side of each merozoite stage transgenic strain’s immunofluorescence panel. The mis-localized signals were marked with arrows. (**J**) Solubility assay detects membrane association of Palm-mutN (mutN) and Palm-mutC (mutC) using different detergents. F, freeze-thaw extraction; T, Triton X-100 extraction; S, SDS extraction. GAPDH and GAPM2 were used as loading controls for F- and S-extractions, respectively.

PbIMC1g has an IMCp domain (aa 29–184) and a C-terminal region (aa 185–297). To assess which domain is needed for proper protein localization, we generated deletion constructs of PbIMC1g for episomal expression in wild-type *P. berghei* (Fig. 3A). Episomal expression of mCherry-tagged full-length PbIMC1g protein (FL) showed a similar localization pattern as PbIMC1g-HA (Fig. 3B). C-terminal deletion (ΔC) also localized the protein to the merozoite periphery, albeit diffused signals in the parasite cytoplasm were also visible (Fig. 3B). In contrast, deletion of the IMCp domain (ΔIMCp) completely abolished the peripheral localization pattern of the protein (Fig. 3B). To determine whether these deletion mutants were SPN-associated, we performed the detergent extraction-based protein solubility assay. The FL protein remained SPN-bound (only in the SDS-soluble fraction), while ΔC was detected in both soluble (freeze-thaw) and SPN-bound fractions. In contrast, ΔIMCp was exclusively found in the soluble fraction (Fig. 3C; Fig. S3A through C). Therefore, the IMCp domain and, to a lesser extent, the C-terminal region of PbIMC1g protein are needed for its efficient IMC association.

### Palmitoylation of PbIMC1g is required for its IMC recruitment and is essential for gametocytogenesis

Previous studies have shown that protein palmitoylation, catalyzed by aspartate–histidine–histidine–cysteine (DHHC) palmitoyl acyltransferases in *Plasmodium*, plays a vital role in targeting proteins to the IMC and mediating protein-protein interactions (32–34). PbIMC1g has seven predicted palmitoylation sites at Cys residues 2, 10, 11, 233, 238, 255, and 296 (Fig. S3D). To determine whether PbIMC1g is palmitoylated in *P. berghei*, we collected lysates from schizonts and ookinetes and labeled them with a clickable alkynyl palmitic acid (Alk-C16) (35). We observed palmitoylation signals in both stages of PbIMC1g^HA^ parasites (Fig. 3D; Fig. S3E) and detected specific palmitoylation of PbIMC1g-HA (Fig. 3E; Fig. S3E and F). Additionally, treatment of PbIMC1g^HA^ schizont with 100 µM of 2-bromopalmitate (2-BP), an inhibitor of protein palmitoylation (36), entirely impaired the proper localization of PbIMC1g in the schizont stage, with the fluorescent signals of PbIMC1g becoming more diffused and losing IMC association (Fig. 3F and G). Inhibition of palmitoylation has been shown to disrupt parasite morphology (37). We found that 2-BP treatment inhibited ookinete conversion, with over 98% of the parasites retained at the retort stage (Fig. 3F). However, the localization profile of PbIMC1g in ookinetes was unaffected, suggesting that palmitoylation does not affect the protein’s IMC targeting in ookinetes (Fig. 3F and G). Together, these results confirmed that PbIMC1g is a palmitoylated protein and implied an effect of palmitoylation on the IMC association of this protein in the schizont stage.

To evaluate the importance of the predicted PbIMC1g palmitoylation sites in proper IMC targeting, we generated two PbIMC1g expression constructs in which the cysteine residues at N-terminus (C2, C10, and C11) and C-terminus (C233, C238, C255, and C296) were mutated to alanine, respectively (Fig. 3H). Disruption of the palmitoylation sites with both constructs resulted in noticeable mis-localization of the protein to the parasite cytoplasm in schizonts (Fig. 3I). However, the localization pattern of Palm-mutN and Palm-mutC at the ookinete stage was not altered (Fig. 3I). Consistent with the ookinete-stage IFA results, immunoblotting revealed that the Palm-mutN and Palm-mutC proteins were presented in the SPN-bound (SDS) fraction of ookinete-stage lysates (Fig. 3J; Fig. S3G).

**Fig 4:**
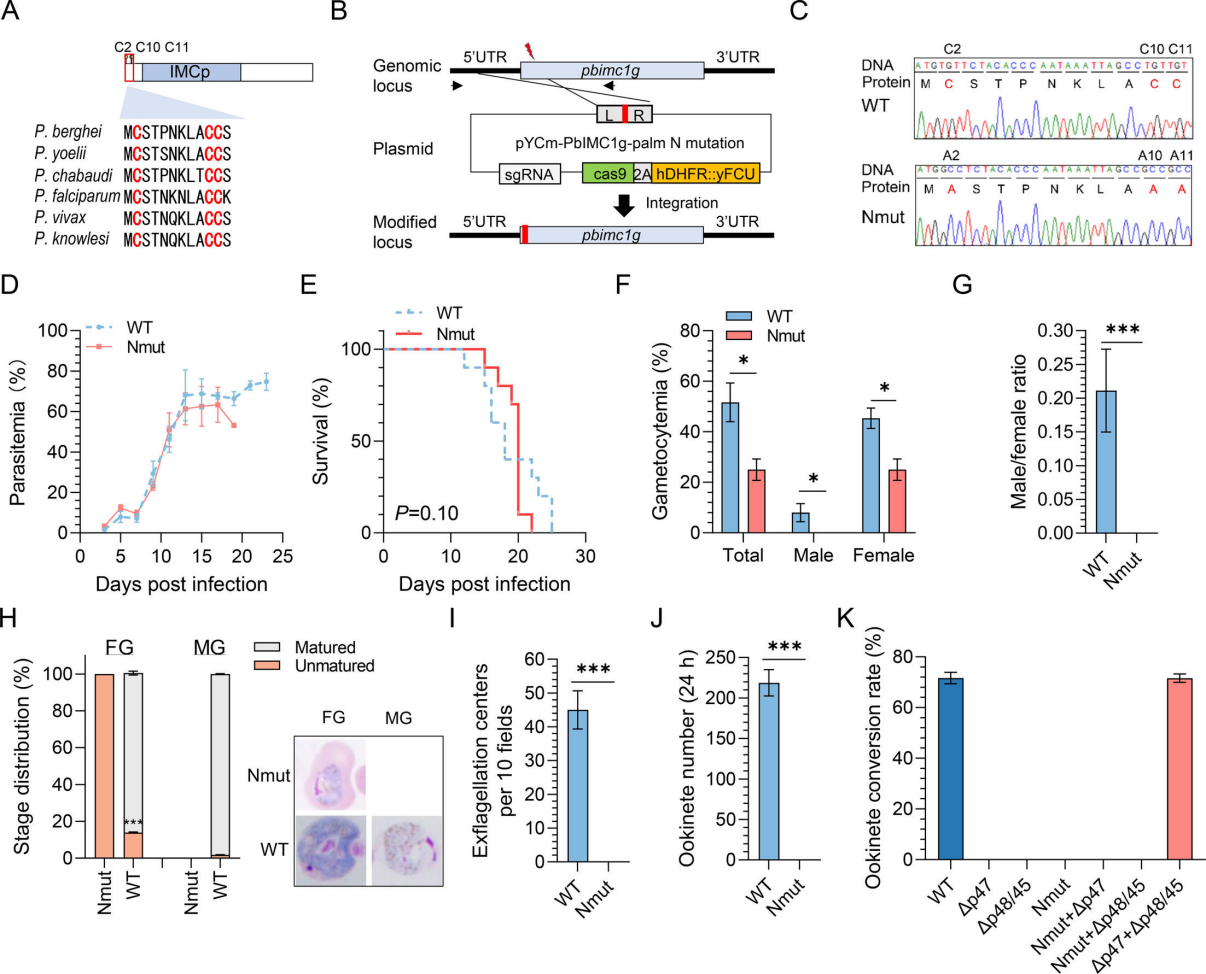
Editing of PbIMC1g to generate a C2/10/11A mutant strain (Nmut). (**A**) Schematic illustrating PbIMC1g with putative N-terminal palmitoylation sites that have been predicted by CSS-Palm 4.0 software. C2, C10, and C11 are shown in red-colored letters. (**B**) Illustration depicting the strategy to mutate C2, C10, and C11 of PbIMC1g to alanine. The location of PCR primers used for genotyping is indicated in the figure. (**C**) A PCR product encompassing the modified locus was sequenced. An image of the chromatogram reveals successful editing of the locus of interest that resulted in the C2/10/11-to-A mutation. (**D**) Comparison of the proliferation curves of wild-type *Pb*. ANKA (WT) and Nmut parasites. Parasitemia in Nmut and WT parasite-infected mice was determined daily by light microscopy of Giemsa-stained blood smears. Data are the mean ± SD of three independent experiments (*n* = 10 mice/group). Statistical significance between WT and Nmut parasites was determined by two-tailed Student’s t-test. (**E**) The effects of N-terminal palmitoylation site mutation on the survival of infected mice. Kaplan-Meier survival curves of mice infected with the WT and Nmut parasites. Data are the mean ± SD of three independent experiments (*n* = 10 mice/group). (**F**) Gametocytemia of the WT and Nmut parasites at 3 days post-infection (dpi). (**G**) Male/female ratios of the WT and Nmut parasites at 3 dpi. (**H**) Stage distribution of WT and Nmut gametocyte at 3 dpi. (**I**) The number of exflagellation centers per 10 fields in WT and Nmut parasites at 3 dpi. (**J**) Ookinete numbers per well at 24 h post *in vitro* culture assay. (**K**) Cross-fertilization of Nmut line with Δp47 and Δp48/45. All bar graphs in this figure show mean ± SD from three biological replicates. *, *P* < 0.05; ***, *P* < 0.001.

Compared to the palmitoylation prediction core of the C-terminus, the N-terminal of PbIMC1g contains higher predicted scores of palmitoylation sites. To better understand how palmitoylation regulates PbIMC1g function, we generated a transgenic strain (Nmut) with N- (C2, C10, and C11) palmitoylation sites mutated to alanines using CRISPR-cas9 genome editing technology (Fig. 4A and B). The successful genomic editing of N-terminal palmitoylation sites in the Nmut strain was confirmed by sequencing (Fig. 4C). The Nmut parasites proliferated, with a parasitemia of ~63.4% at day 17 post-infection (dpi), comparable to WT *P. berghei* (Fig. 4D). Meanwhile, the Nmut strain-infected group also exhibited a comparable survival span to the WT group, indicating that N-terminal palmitoylation of PbIMC1g is not required for its function in asexual-stage proliferation (Fig. 4E). However, the Nmut strain exhibited a significant 100%, 44.8%, and 100% reduction in male and female gametocytemia and male/female gametocyte ratio at 3 dpi (Fig. 4F and G). No male gametocytes and only a few unmatured female gametocytes were observed in the Nmut strain at 3 dpi (Fig. 4H). Furthermore, neither exflagellation center formation nor ookinete formation was observed in the Nmut strain at 3 dpi (Fig. 4I and J). Cross-fertilization assay using parasite lines defective in either female (Δp47) or male gametes (Δp48/45) revealed that both male and female gametes’ function was impaired in Nmut parasites (Fig. 4K). Therefore, we conclude that the palmitoylation sites are necessary for incorporating PbIMC1g into the IMC in the schizont stage and are essential for parasites’ gametocytogenesis.

### PbIMC1g is required for correct cytokinesis during schizogony

Multiple attempts to knock out *pbimc1g* failed, as shown the same for PbIMC1c (38), suggesting that it is essential for asexual parasite replication. Therefore, we generated the PbIMC1g^cKD^ parasite by tagging the C-terminus of the endogenous *pbimc1g* gene with a triple HA tag and a dihydrofolate reductase (DHFR)-based destabilizing domain (3×HA-DDD) for conditional KD of PbIMC1g using the DDD system (39, 40) (Fig. S2C). Successful modification of the *pbimc1g* locus in *P. berghei* was confirmed by genotyping PCR (Fig. S2D). Immunoblotting with blood-stage PbIMC1g^cKD^ parasite extracts detected a band of ~70 kDa, larger than the expected size of PbIMC1g-HA-DDD protein, 59.4 kDa (Fig. S2D). After withdrawing trimethoprim (TMP) for 3 days, which stabilized the PbIMC1g-HA-DDD fusion protein, we observed a 73.8% KD of PbIMC1g-HA-DDD protein in the immunoblot with the anti-HA mAb, compared to the steady-state protein level (1 mg/mL TMP) (Fig. S2E). Notably, TMP is an antimalarial agent; thus, an overdose supplement of this drug will also inhibit parasite growth, which would result in decreased PbIMC1g-HA-DDD, as we detected in the 2 mg/mL TMP group (Fig. S2E). PbIMC1g depletion after TMP withdrawal showed a time-dependent manner, with a loss of ~45% protein within 1 h (Fig. S2F). IFA with the anti-HA mAb also confirmed a reduction of ~75% of the fluorescence signal in most parasites within 2 h after TMP withdrawal (Fig. S2G).

**Fig 5:**
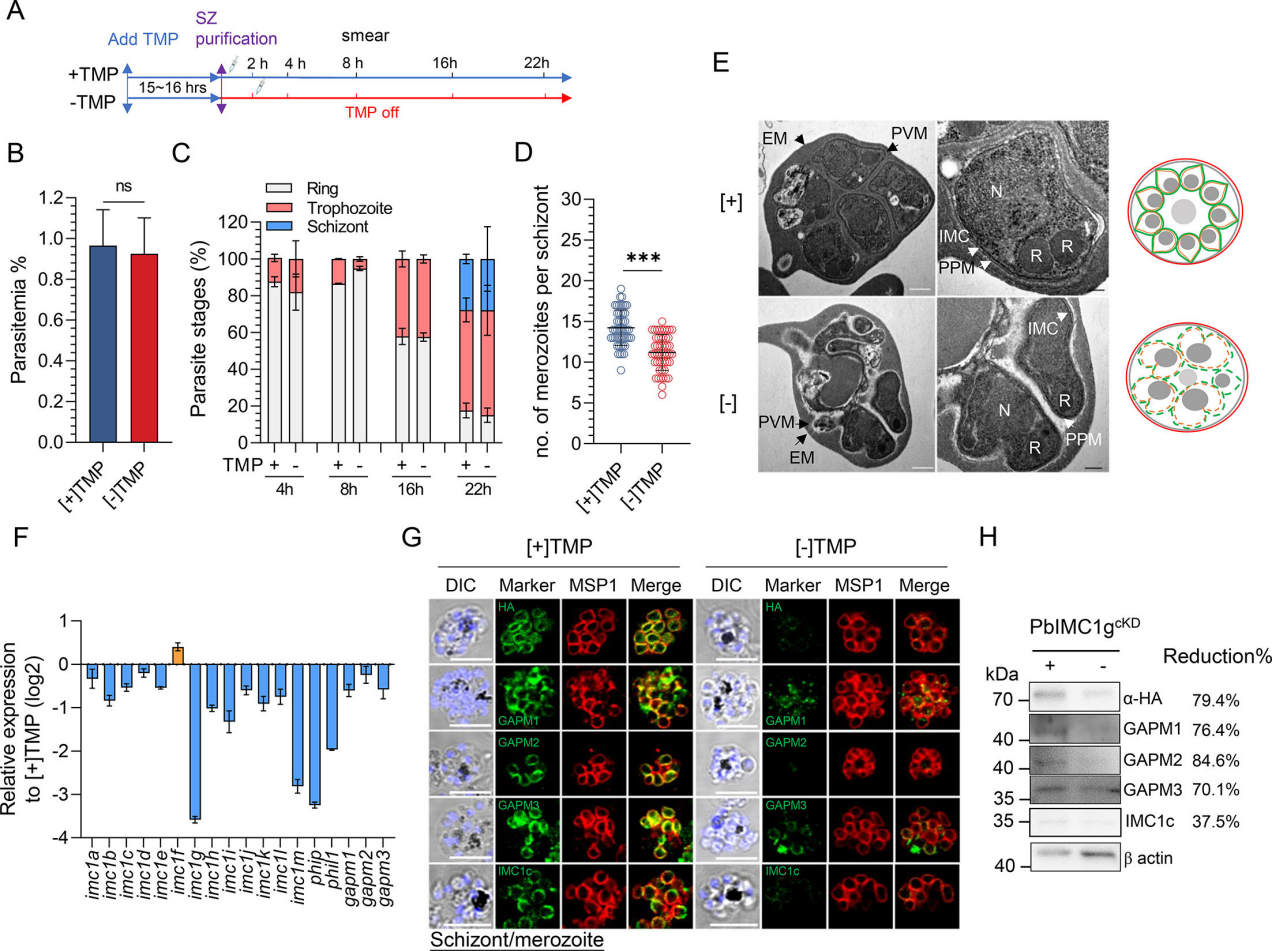
PbIMC1g is required for asexual stage cytokinesis. (**A**) Schematic diagram outlining the methodology used for the *in vivo* assay of PbIMC1g depletion on merozoite invasion. (**B**) Parasitemia of PbIMC1g KD parasite-infected mice at 4 hpi. [+] TMP, PbIMC1g^cKD^ schizont injected directly after purification; [−] TMP, purified PbIMC1g^cKD^ schizont injected after 2-h incubation with medium without additional TMP supplement. ns, not significant. (**C**) The intraerythrocytic development of PbIMC1g-depleting parasites. PbIMC1g^cKD^ schizonts (10^8^) were purified and intravenously injected into mice. The infected mice were supplied with ([+]) or without ([−]) 1 mg/mL of TMP in drinking water. After 4, 8, 16, and 22 h post-infection, the percentages of rings (grey), trophozoites (pink), and schizonts (blue) were determined. Data represents mean ± SD of two independent experiments in duplicates (Tukey’s multiple comparison tests were applied between [+] and [−] TMP groups at the same time points). (**D**) Number of merozoites per schizont obtained after *in vitro* culture of PbIMC1g^cKD^ [+]/[−] TMP parasites. Data represents mean ± SD of two independent experiments (*n* = 60). ***, *P* < 0.001 (Mann–Whitney *U* test). (**E**) TEM images of matured schizont stage PbIMC1g^cKD^ [+]/[−] TMP parasites from *in vitro* schizont cultures. In both images, the different membranes are indicated as follows: erythrocyte (EM, black arrowhead), parasite vacuole (PVM, black arrowhead), parasite plasma (PPM, white arrow), and inner membrane complex (IMC, white arrow). N, nucleus; R, rhoptry. Representative of two experiments. Experiment 1, *n* = 20; experiment 2, *n* = 15. Scale bars, 500 nm (white), 200  nm (black). Schematics of IMC and PPM formation during schizogony of PbIMC1g^cKD^ [+]/[−] TMP parasites *in vitro* are shown in the lower panel. (**F**) Real-time PCR analysis of inner membrane complex located proteins in PbIMC1g^cKD^ [+]/[−] TMP parasites. (**G**) IFA of PbIMC1g^cKD^ schizonts cultured in the presence ([+]) or absence ([−]) of 1 µM TMP. The IFA images were detected using anti-HA, anti-GAPM1, anti-GAPM2, anti-GAPM3, and anti-IMC1c antibodies. Scale bar, 5 µm. (**H**) Western blot analysis of expression levels of PHIL1 complex in PbIMC1g KD parasites. The immunoblot membrane was probed with anti-HA, anti-GAPM1, anti-GAPM2, anti-GAPM3, and anti-IMC1c antibodies, respectively. +, [+] TMP; −, [−] TMP. β-Actin was used as a loading control. Relative band intensity (normalized to the signal intensity of β-actin) of each protein in [−] TMP compared to [+] TMP that indicates that the reduction of protein expression level was shown in the right panel.

To clarify the function of PbIMC1g in asexual blood-stage development, we first investigated whether PbIMC1g KD affected merozoite invasion of host RBCs. Purified PbIMC1g^cKD^ schizonts were cultured *in vitro* in the presence or absence of 1 µM of TMP for 2 h and then injected into BALB/c mice (Fig. 5A). Similar parasitemias were observed in infected mice at 4 hpi in the [+] and [−] TMP treatment groups, suggesting that PbIMC1g was not essential for merozoite invasion (Fig. 5B). We then investigated the parasite’s intraerythrocytic development with the KD of PbIMC1g. Both [+] and [−] TMP parasites showed ~42% trophozoites at 16 hpi, and ~27% schizonts at 22 hpi, indicating that PbIMC1g KD did not delay the intraerythrocytic growth of *P. berghei* (Fig. 5C).

To test the effect of PbIMC1g KD in the late schizont stage, PbIMC1g^cKD^ parasite-infected blood at 3 dpi was used for *in vitro* culture under [+] or [−] TMP supplement. Although the [−] TMP parasites progressed through the trophozoite stage, replicated the DNA, and formed schizonts, they produced significantly fewer merozoites per schizont (11.2 ± 0.3) than the [+] TMP parasites (14.2 ± 0.3), suggesting defects in cytokinesis (Fig. 5D). To gain deeper insights into the morphological changes in the [−] TMP parasites, we examined the mature schizonts by transmission electron microscopy (TEM). Although both [+] and [−] TMP schizonts exhibited rhoptries and nuclei, the [+] TMP schizonts displayed clearer and more uniform segregation of the merozoites, surrounded by a continuous parasite plasma membrane (PPM) and IMC (Fig. 5E; Fig. S4A). In contrast, [−] TMP schizonts showed disorganized and insufficiently segregated merozoites with disrupted PPM and IMC, indicating incomplete cytokinesis and formation of IMC (Fig. 5E; Fig. S4A). This is consistent with the role of IMC1 proteins in maintaining cytoskeleton integrity in apicomplexan parasites. Defects upstream of cytokinesis upon PbIMC1g KD were further supported by the marked reduction in the transcript level of several IMC proteins, such as IMC1 family members (*imc1a-e* and *imc1g-m*) and PHIL1 complex component (*phip*, *phil1*, *gapm1*, *gamp2*, and *gapm3*) in PbIMC1g-depleting parasites (Fig. 5F).

Since PbIMC1g interacts with the PHIL1 complex, and transcripts of these genes were also affected, we inspected the expression of PHIL1 components in PbIMC1g KD schizonts by IFA and immunoblotting. By using antisera against PHIL1 components (Fig. S5), we found that the localization patterns of GAPM1, GAMP2, GAPM3, and IMC1c were substantially altered, and their expression levels were reduced by 37.5%–84.6% in [−] TMP schizonts, suggesting that PbIMC1g depletion affected both the localization and expression of PHIL1 components (Fig. 5G and H). Together, these observations showed that PbIMC1g is important for asexual stage schizogony.

### PbIMC1g KD leads to dysregulated expression of genes involved in male gametogenesis

**Fig 6:**
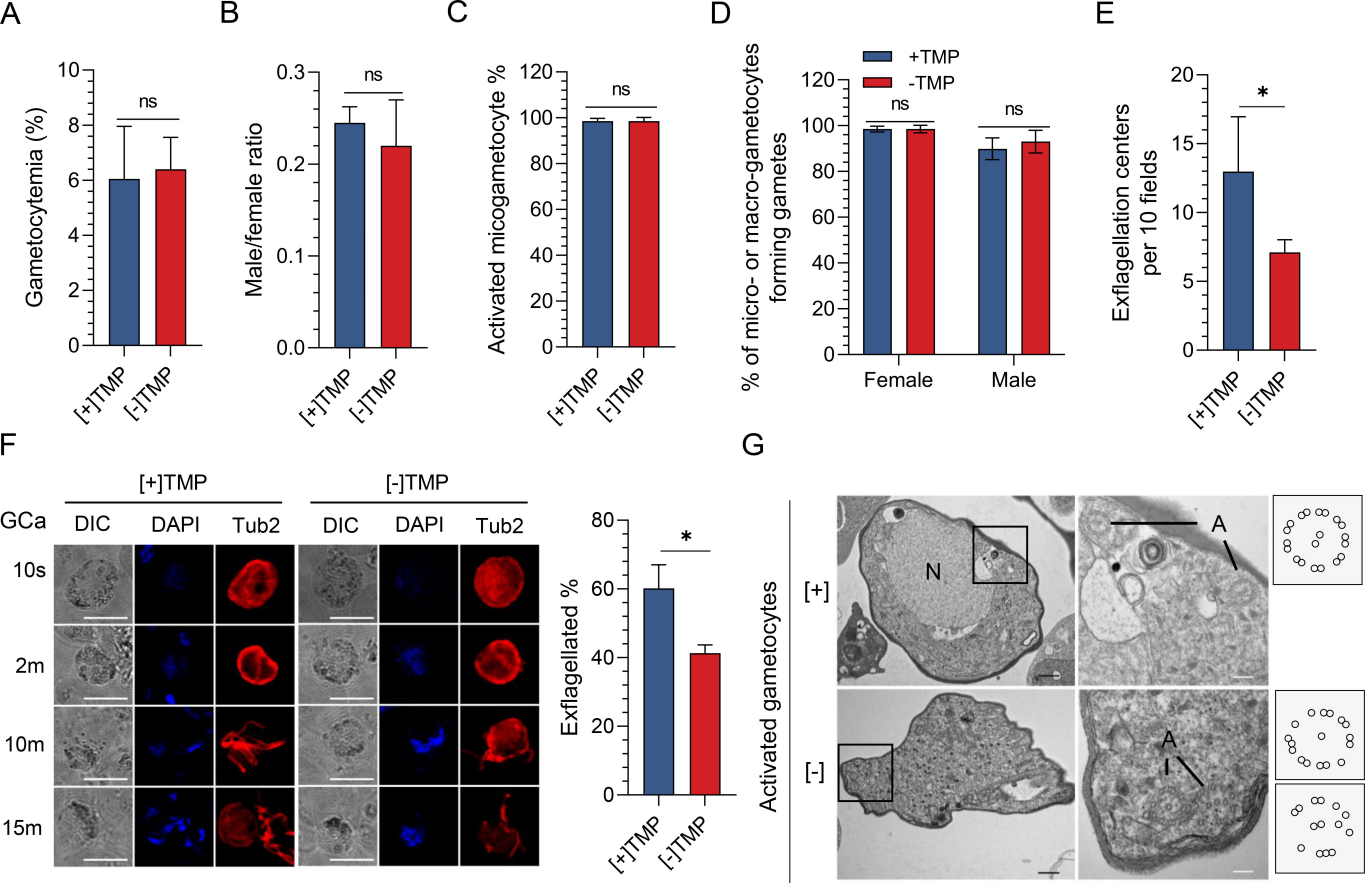
PbIMC1g is required for male gametogenesis. (**A**) Gametocytemia of the PbIMC1g^cKD^ parasites in the absence (−TMP) or presence (+TMP) of 1 mg/mL TMP at 3 days post-infection (dpi). (**B**) Male/female ratios of the PbIMC1g^cKD^ [+]/[−] TMP parasites at 3 dpi. (**C**) The percentage of activated PbIMC1g^cKD^ [+]/[−] TMP microgametocyte at 3 dpi. (**D**) The proportions of macrogametocytes or microgametocytes forming gametes (%) in PbIMC1g^cKD^ [+]/[−] TMP parasites at 3 dpi. Gametocytes, Ter119 positive and α-tubulin II/G377 positive; gametes, Ter119 negative and α-tubulin II/G377 positive. (**E**) Comparison of exflagellation centers formation from PbIMC1g^cKD^ [+]/[−] TMP parasites. The numbers of exflagellation centers (mean ± SD) were determined from two independent experiments. (**F**) IFA images of PbIMC1g^cKD^ [+]/[−] TMP male gametocytes showing the Tubulin II (tub2, red) at different time points after activation (left panel). Scale bars, 5 µm. The quantified percentages of exflagellated PbIMC1g^cKD^ [+]/[−] TMP parasites after activation are shown in the right panel. (**G**) TEM micrographs of male gametogenesis of PbIMC1g^cKD^ [+]/[−] TMP parasites at 8 mpa. N, nucleus; A, axonemes. Scale bars, 500 nm (black), 200  nm (white). Schematics of axonemes of PbIMC1g^cKD^ [+]/[−] TMP parasites at 8 mpa were shown in the right panel. Each group examined 40 parasites (*n* = 40) for TEM analysis. Statistical analysis for panels **A–F** was done using the Student’s *t*-test. ns, not significant; *, *P* < 0.05.

We further used the PbIMC1g^cKD^ line to analyze PbIMC1g’s functions in sexual development and transmission to mosquitoes. TMP was withdrawn from 0 dpi, and gametocytogenesis was evaluated at 3 dpi. Compared to the [+] TMP group, [−] TMP did not affect gametocytemia or the male-to-female sex ratio (Fig. 6A and B). Moreover, the proportions of macrogametocyte activation and macrogamete formation were comparable between the [+] and [−] TMP parasites (Fig. 6C and D). However, the number of exflagellation centers in [−] TMP parasites (7.1 ± 0.9 per 10 fields) was significantly reduced compared to [+] TMP parasites (13.0 ± 4.0 per 10 fields) (Fig. 6E). When a time-course analysis of activated gametocytes was performed using α-tubulin II, we observed a 16.9% reduction in exflagellated male gametocytes in [−] TMP parasites (*P* < 0.05, Fig. 6F). TEM analysis of male gametocytes at 8 min post-activation revealed 70.8% of axonemes with disorganized microtubules (9+1 or 5+2) in [−] TMP microgametocytes compared to the normal axonemes with a 9+2 organization in [+] TMP microgametocytes (Fig. 6G; Fig. S4B and C).

**Fig 7:**
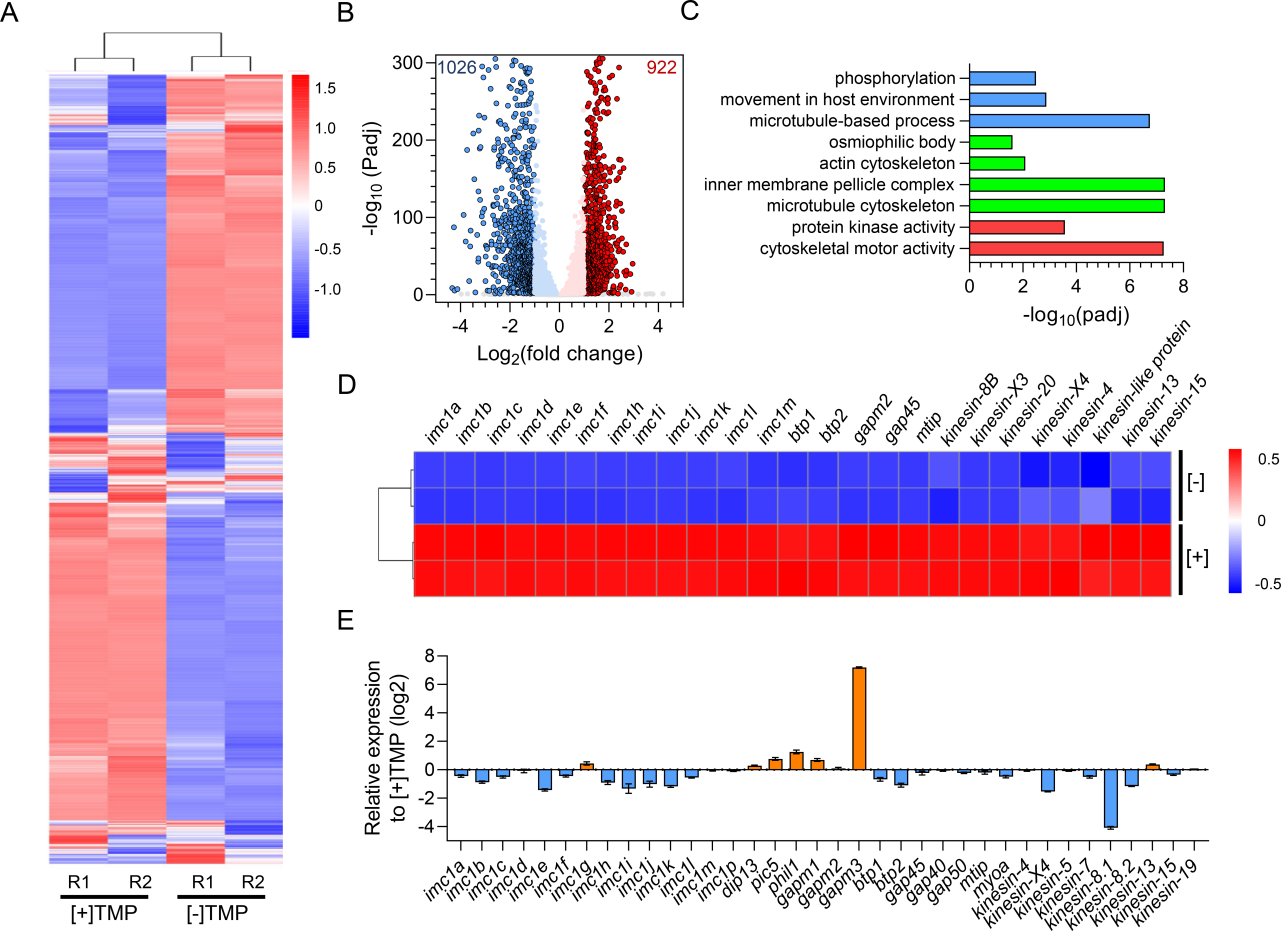
Transcriptome analysis for activated gametocyte of PbIMC1g knockdown parasites by RNA-seq. (**A**) Hierarchical clustering of all genes with significant changes in expression. The normalized FPKM values are shown on the vertical axis and strain information ([+] TMP, PbIMC1g protein with normal expression level; [−] TMP, PbIMC1g knockdown parasites) on the horizontal axis. Clustering is based on Spearman correlation coefficients and plotted using an R program. Duplicates from each experimental group clustered independently (upper dendrogram). Refer to Table S1A for the data sets used to generate this figure. (**B**) Volcano plot showing the extent and significance of upregulated (red) and downregulated (blue) genes in the [−] TMP parasites compared to [+] TMP (absolute log_2_ fold change  > 1). Refer to Table S1B. (**C**) Gene ontology enrichment analysis of significant twofold regulated genes in [−] TMP parasites compared to [+] TMP parasites. GO terms representing the biological processes (BP), cellular component (CC), and molecular function (MF) are presented in blue, green, and red bars, respectively. Refer to Table S1C. (**D**) Heatmaps showing differential expression of selected genes in the PbIMC1g^cKD^ [+]/[−] TMP parasites. Refer to Table S1D. (**E**) qRT-PCR validation of the expression of cytokinesis-related genes in [−] TMP-treated parasites compared with [+] TMP-treated parasites. The up- and downregulated genes are presented in orange and blue bars, respectively. The PbIMC1g^cKD^ [+]/[−] TMP gametocytes were purified using 48% Nycodenz and activated at 25℃ for 15 min. Error bars indicate SD from three biological replicates.

To further understand the molecular basis of the impaired gametogenesis due to PbIMC1g KD, we analyzed the transcriptomes of activated gametocytes of the [+] and [−] TMP parasites by RNA-seq. Correlation analysis demonstrated excellent reproducibility among biological replicates (PCC *R*^2^ = 0.994–1; Table S1A). We identified 1948 transcripts with significantly altered expression (adjusted *P*-value < 0.05 and log_2_ fold change > 1) in the [−] TMP parasites compared to [+] TMP parasites (Fig. 7A; Table S1B), including 922 upregulated and 1026 downregulated genes (Fig. 7B; Table S1C). Gene ontology (GO) enrichment analysis showed that the downregulated transcripts include GO terms associated with cytoskeletons, such as the inner membrane pellicle complex (29 proteins), microtubule cytoskeleton (30 proteins), actin cytoskeleton (10 proteins), and osmiophilic body (6 proteins) (Fig. 7C; Table S1D). These downregulated transcripts include genes encoding IMC proteins, such as IMC1 family members (*imc1a-f*, *imc1h-m*, and *imc1p*), basal complex (*btp1* and *btp2*), kinesins (*kinesin-8B*, *-X3*, *-20*, *-X4*, *-4*, *kinesin-like protein*, *-13*, and *-15*), and glideosome (*gap45*, *gap40*, *gap50*, *mtip*, and *myoa*), indicating that the PbIMC1g KD influenced the cytoskeleton protein expression during gametogenesis (Fig. 7D; Table S1E). Real-time PCR analysis confirmed the downregulated expression of IMC1 family members, basal complex, glidesome, and kinesins (Fig. 7E). Our data indicated that PbIMC1g is required for male gametogenesis in *P. berghei*.

### PbIMC1g KD impairs ookinete conversion

**Fig 8:**
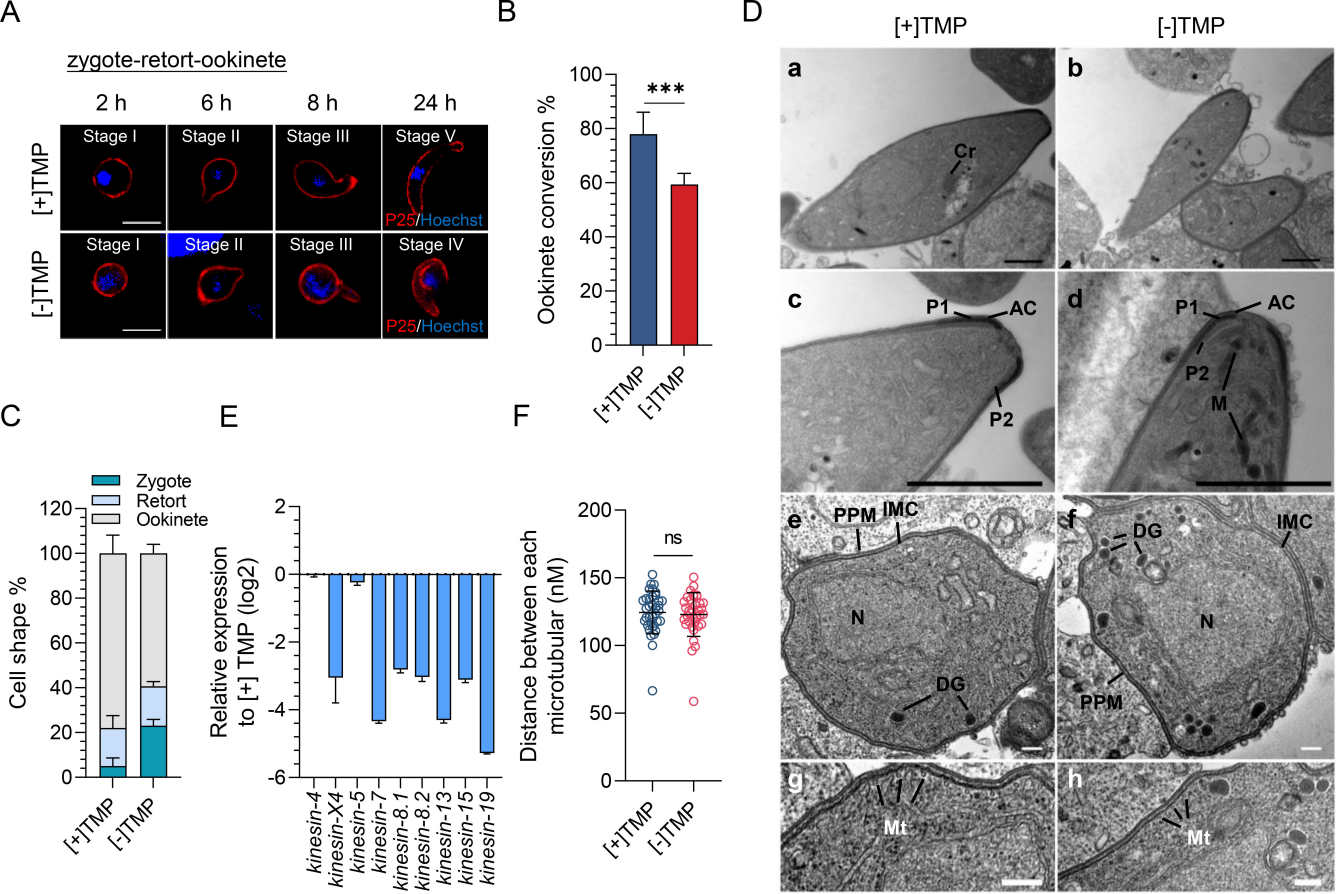
Transmission electron microscopy analysis of PbIMC1g^cKD^ [+]/[−] TMP parasites at the ookinete stage. (**A**) Ookinete developmental stages for PbIMC1g^cKD^ [+]/[−] TMP parasites. Ookinetes were identified using P25 and defined as those cells differentiated successfully into elongated “banana-shaped” forms. DNA is stained with Hoechst dye (blue). Scale bar, 5 µm. (**B**) Ookinete conversion rate (%) in PbIMC1g^cKD^ [+]/[−] TMP parasites. The conversion rate is the percentage of Pbs21-positive parasites successfully differentiated into elongated “banana-shaped” ookinetes. Statistical analysis was done using the Student’s *t*-test. ***, *P* < 0.001. (**C**) Cells of different morphologies (%). (**D**) TEM images of PbIMC1g^cKD^ [+] TMP (**a, c, e, **g) and [−] TMP (**b, d, f, **h) parasites at the ookinete stage. (a–d) Longitudinal section of a crescent-shaped [+] TMP ookinete. AC, apical complex; M, micronemes; Cr, crystalline body; P1, polar ring 1; P2, polar ring 2. (e–h) Cross section through the periphery of the anterior complex of a [+] TMP (**e, **g) and a [−] TMP (**f, **h) parasite showing similar substructure consisting of the outer parasite plasma membrane and the underlying inner membrane complex. N, nucleus; DG, dense granule; Mt, subpellicular microtubules. Scale bars, 1 µm (black), 200  nm (white). (**E**) qRT-PCR validation of the expression of kinesin superfamily genes in [−] TMP-treated parasites compared with [+] TMP-treated parasites. Error bars indicate SD from three biological replicates. (**F**) The distance between each microtubular at the ookinete stage was analyzed using Image J software. *N* = 36 for both [+] and [−] TMP groups. Statistical analysis was done using the Student’s *t*-test. ns, not significant.

Next, we used an *in vitro* culture to examine if PbIMC1g KD affected ookinete development. At 6–8 h, both PbIMC1g^cKD^ [+] and [−] TMP ookinetes developed a short protuberance (stages II–III). At 24 h of *in vitro* culture, 77.9% ± 8.2% of [+] TMP ookinetes transformed into a banana shape, characteristic of mature ookinetes (Fig. 8A and B). In contrast, 59.4% ± 4.1% of [−] TMP parasites showed mature ookinete morphology, whereas 40.6% of parasites were arrested at the zygote (23.1%) and retort (17.5%) stages, respectively (Fig. 8A through C).

We further compared the structures of the ookinetes at 12 h of *in vitro* culture by TEM. Longitudinal sections of the apical complex showed similar ultrastructures between PbIMC1g^cKD^ [+] and [−] TMP ookinetes (Fig. 8D a through d; Fig. S4D a through d). In particular, the anterior polar ring 1 formed by the IMC and a second polar ring representing the initiation site for the subpellicular microtubules were also comparable between [+] and [−] TMP parasites (Fig. 8D). Although PbIMC1g KD led to significantly decreased mRNA levels of microtubule (MT)-based motor proteins, such as the kinesins (*kinesin-4*, *-X4*, *-5*, *-7*, *-8.1*, *-8.2*, *-13*, *-15*, and *-19*) in [−] TMP parasites at 2 h of ookinete cultures (Fig. 8E), IMC was distributed along the whole-cell periphery in PbIMC1g-KD parasites, and both [+] and [−] TMP parasites showed even distribution of microtubules in the IMC (Fig. 8D and F; Fig. S4D e and f). Together, these results suggest that PbIMC1g is not necessary for subpellicular microtubule organization in developing ookinetes.

### PbIMC1g KD affects ookinete motility and mosquito transmission

**Fig 9:**
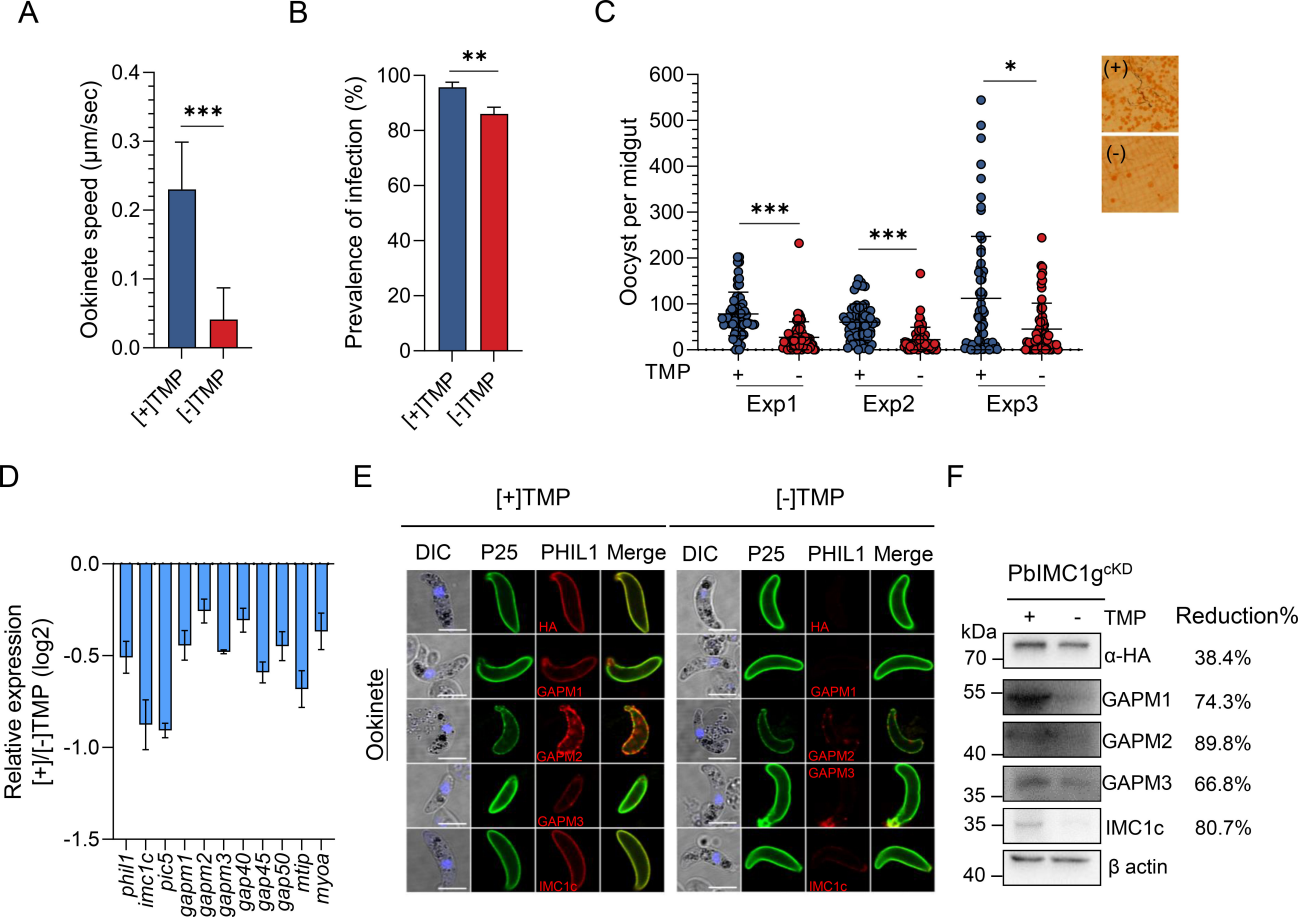
Effect of PbIMC1g knockdown on ookinete motility and infectivity. (**A**) Effect of PbIMC1g KD on the average gliding speed of mature ookinetes in Matrigel. The speeds of PbIMC1g^cKD^ [+]/[−] TMP ookinete were expressed in μm/s. Images were captured every 10 s over a 30-min period. The plot is based on pooled data from two independent experiments and >10 ookinetes analyzed. Statistical analysis was done using the Student’s *t*-test. ***, *P* < 0.001. (**B**) Prevalence of infection of PbIMC1g^cKD^ [+] TMP parasite-infected mosquitoes. **, *P* < 0.01 (**C**) Direct mosquito feeding assay (DFA) in mice infected with the PbIMC1g^cKD^ [+] TMP parasites followed by sucrose feeding in [+]/[−] TMP conditions at 24 h post-feeding. Data points represent midgut oocyst numbers of individual mosquitoes in each group. Results from three independent experiments are shown. Error bars indicate mean ± SD (*n*  =  3). Statistical significance was determined using the Mann–Whitney *U* test. *, *P* < 0.05; ***, *P* < 0.001. (**D**) Real-time PCR analysis of PHIL1 complex and glideosome proteins in PbIMC1g^cKD^ [+]/[−] TMP parasites. (**E**) Immunofluorescence analysis of PbIMC1g^cKD^ [+]/[−] TMP ookinetes. The IFA images were detected using anti-HA, anti-GAPM1, anti-GAPM2, anti-GAPM3, and anti-IMC1c sera, respectively. Scale bar, 5 µm. (**F**) Immunoblotting of expression levels of PHIL1 complex in PbIMC1g-depleting parasites. The immunoblot membrane was probed with anti-HA, anti-GAPM1, anti-GAPM2, anti-GAPM3, and anti-IMC1c sera, respectively. β-Actin was used as a loading control. Relative band intensity (normalized to the signal intensity of β-actin) of each protein in [−] TMP compared to [+] TMP that indicates the reduction of protein expression level is shown in the right panel.

Since ookinete is a motile and invasive stage, we further examined the motility of morphologically normal [+] TMP and [−] TMP ookinetes. The [+] and [−] TMP ookinetes at 24 h of ookinete culture were embedded in Matrigel, and their motility was observed using a time-lapse assay (28). The [+] TMP ookinetes possessed normal corkscrew-like movement at an average speed of ~0.23 µm/s (range 0.14–0.32 μm/s), whereas the [−] TMP ookinetes were much slower, with an average speed 0.04 µm/s (range 0–0.09 μm/s). Besides, the trajectories of the [−] TMP ookinetes were more linear, indicating that the gliding motility of [−] TMP ookinetes was impaired (Fig. 9A; Movies S1 and S2).

To assess the infectivity of PbIMC1g KD ookinetes, *Anopheles stephensi* mosquitoes were allowed to feed on mice infected with [+] TMP parasites, followed by [+] or [−] TMP supplementation provided in 10% (wt/vol) glucose solution. Counting oocysts in mosquito midgut on 10 dpi revealed a significantly lower prevalence of infection in [−] TMP-treated mosquitoes compared to [+] TMP-treated mosquitoes (86% vs 95.7%, *P* < 0.01; Fig. 9B). Furthermore, the [−] TMP-treated mosquitoes had a 52.6% reduction in oocyst density compared to [+] TMP-treated mosquitoes, indicating that PbIMC1g KD may have adversely affected ookinete infectivity (Fig. 9C; Table 1).

**TABLE 1:** Transmission of PbIMC1g-depleted parasites in *An. stephensi^a^*

Parameter	[+] TMP			[−] TMP		
	Expt 1	Expt 2	Expt 3	Expt 1	Expt 2	Expt 3
Mosquito-infected/dissected	60/62	60/62	58/62	54/62	59/62	62/62
Prevalence of infection (%)*^b^*	96.8	96.8	93.5	88.7	85.5	83.9
Mean prevalence (%)			95.7			86.0
Reduction in prevalence (%)*^c^*						9.7**
Oocyst intensity*^d^*	78.2	60.2	112.0	27.4	22.0	45.0
SEM*^e^*	6.1	5.0	17.1	4.3	3.4	7.2
Mean oocyst intensity			83.5			31.5
Reduction in oocyst intensity (%)*^f^*						52***

^*a*^ Expt, experiment.

^*b*^ The prevalence of infection was calculated by the number of mosquitoes with oocysts/total mosquitoes dissected in each group × 100%.

^*c*^ The percent reduction of prevalence was calculated as % mean prevalence_[+] TMP_ − % mean prevalence_[−] TMP_; Fisher’s exact test; **, *P* < 0.01.

^*d*^ Mean number of oocysts per mosquito midgut.

^*e*^ Standard error of the mean.

^*f*^ The percent reduction in oocyst intensity was calculated as (mean oocyst intensity_[+] TMP_ − mean oocyst intensity_[−] TMP_)/mean oocyst intensity_[+] TMP_ × 100%; Mann–Whitney *U* test; ***, *P* < 0.001.

The glideosome regulates ookinete motility, and the PHIL1 complex firmly anchors the glideosome to the IMC by interacting with cytoskeletal alveolins (10, 41). Real-time PCR analysis revealed a significant reduction in mRNA levels of PHIL1 complex (*phil1*, *imc1c*, *pic5*, *gapm1*, *gapm2*, and *gapm3*), as well as the glideosome proteins (*gap40*, *gap45*, *gap50*, *myoa*, and *mtip*) in [−] TMP parasites at 2 h of ookinete culture, suggesting PbIMC1g regulates the expression levels of PHIL1 complex and glideosome components in ookinetes (Fig. 9D). To ascertain if the PHIL1 components were involved in ookinete motility in PbIMC1g KD parasites, the characteristic banana-shaped ookinete at 24 h from [−] TMP *in vitro* ookinete cultures was examined. We found that the subcellular localization and expression levels of PHIL1 components, including IMC1c, GAPM1, GAPM2, and GAPM3, were altered and significantly reduced (38.4%–89.8%), indicating that PbIMC1g is responsible for the localization and stability of PHIL1 complex (Fig. 9E and F). Together, these results showed that PbIMC1g contributes to the gliding motility of ookinetes.

### Allele replacement indicates functional conservation of IMC1g in *Plasmodium*

The significant sequence homology among *Plasmodium* IMC1g members suggests they are functionally conserved. To test this hypothesis, we replaced the *pbimc1g* coding sequence with *P. vivax imc1g* ortholog (PVX_079955), while simultaneously tagging the PvIMC1g with a 2×Myc tag (Fig. S6A). Successful allele replacement was confirmed by diagnostic PCR (Fig. S6B), and the PvIMC1g-Myc protein expression in a transgenic *P. berghei* parasite line (PvIMC1g^TR^) was confirmed by western blotting using the anti-Myc mAb (Fig. S6C). The PvIMC1g-Myc protein exhibited a similar localization profile as in the PbIMC1g^HA^ transgenic parasite (Fig. S6D). Similarly, the PvIMC1g-Myc protein was predominantly presented in the SDS fraction and interacted with the PbIMC1c protein, suggesting that the protein targeting and protein-protein interaction of IMC1g are conserved in *Plasmodium* (Fig. S6E and F).

**Fig 10:**
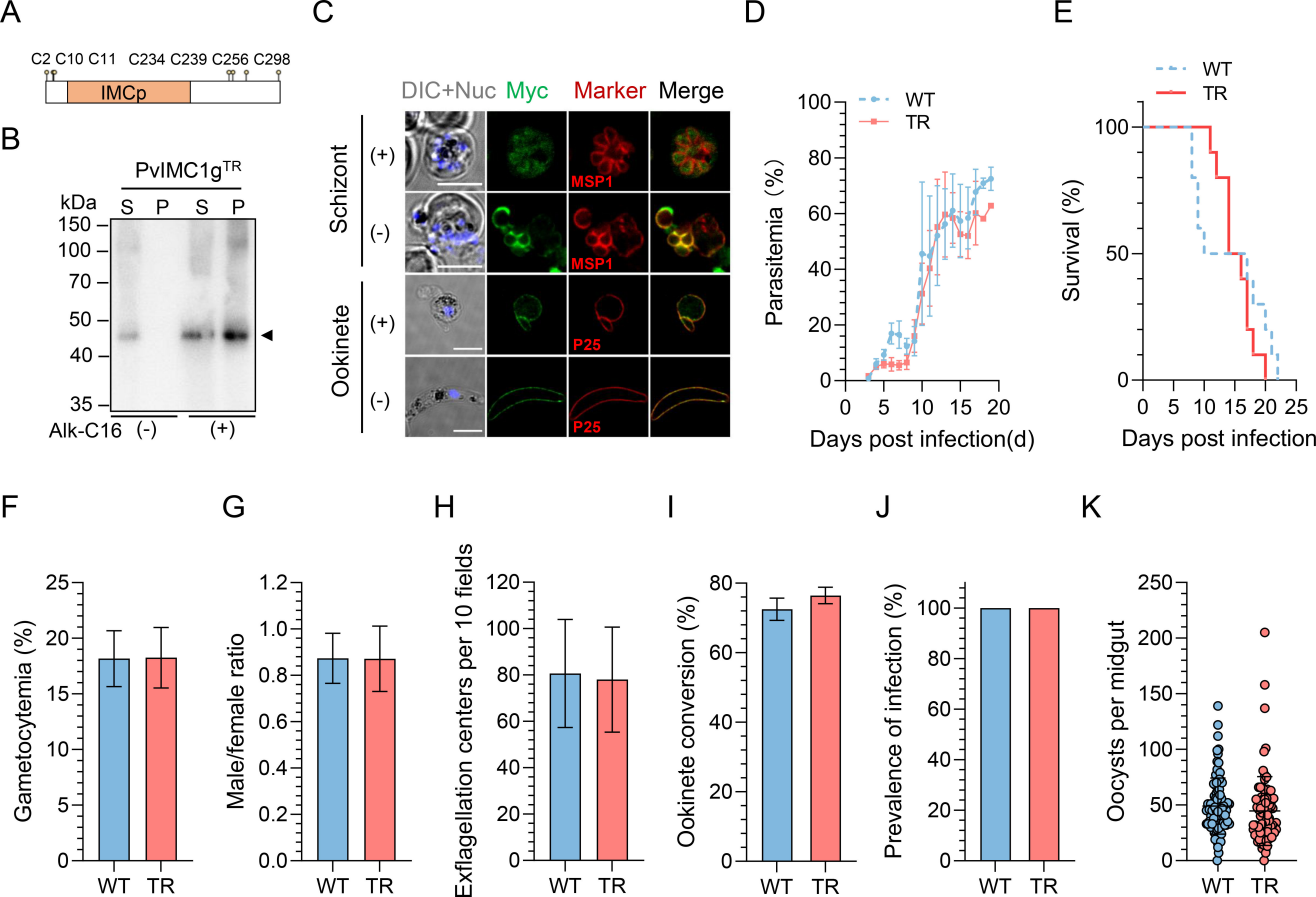
Phenotypic analysis of the PvIMC1g^TR^-transgenic parasites in asexual and sexual stages. (**A**) Schematic figure of predicted palmitoylated sites on PvIMC1g protein. (**B**) Click chemistry method detecting palmitoylation of PvIMC1g-Myc protein in PvIMC1g^TR^ parasites at ookinete stages. The PbIMC1g^HA^ ookinetes were metabolically labeled with (+) or without (−) 25 µM of Alk-C16 for 4 h, biotinylated by click reaction, followed by precipitation with streptavidin agarose resin. The captured palmitoylated proteins were analyzed by western blotting using anti-Myc mAb. S, supernatant; P, pellet. The arrowhead indicates PvIMC1g-Myc protein. (**C**) IFA of 100 µM 2-BP-treated PvIMC1g^TR^ parasites at schizont and ookinete stages. Parasites were co-stained with anti-Myc mAb and anti-MSP1 (schizont) or anti-P25 (ookinetes) sera. The nuclei (nuc) were stained with DAPI. Scale bar, 5 µm. (**D**) Growth curves of the *P. berghei* ANKA (wild type [WT]) and transgenic PvIMC1g^TR^ (TR) parasites. Each mouse was inoculated with 1 × 10^6^ iRBCs, and parasitemia was monitored daily. Data are the mean ± SD of three independent experiments. (**E**) Kaplan-Meier survival curve of mice infected with the WT and TR parasites. Each group has five mice. The graph is representative of three independent experiments. (**F**) Gametocytemia of WT and TR transgenic parasites at 3 dpi. (**G**) Male/female gametocyte ratios of WT and TR parasites at 3 dpi. (**H**) Exflagellation centers formation in the WT and TR parasites at 3 dpi. (**I**) Ookinete conversion rates (%) of the WT and TR parasites. Data were representative from three independent experiments for figures (**F–I**). *n* = 5 each. (**J**) Prevalence of infection of WT and TR parasite-infected mosquitoes. (**K**) Midgut oocysts in mosquitoes infected with WT and TR strain at 10 dpi. Each dot indicates the oocyst number of a mosquito, while the horizontal bars indicate the mean  ±  SD. The graph shows two independent feeding experiments. For each feeding experiment, 40 mosquitoes were dissected for the WT and TR parasites, respectively.

GPS-Palm software predicted PvIMC1g as a palmitoylated protein, and the cysteine residues C2, C10, C11, C234, C239, C256, and C298 of PvIMC1g are evolutionarily conserved among different *Plasmodium* species (Fig. 10A; Fig. S3D). Indeed, we detected palmitoylation in PvIMC1g-Myc protein using the click chemistry method (Fig. 10B). Similarly, 2-BP treatment significantly impaired the proper localization of PvIMC1g-Myc protein in schizont-stage parasites. Although 2-BP treatment blocked ookinete development, the fluorescence signals of PvIMC1g-2Myc remained in the periphery of parasites (Fig. 10C). These results confirmed that IMC1g is palmitoylated in *Plasmodium* spp.

Compared to the WT *P. berghei* parasites, the transgenic PvIMC1g^TR^ parasite showed similar asexual blood-stage growth in infected mice (Fig. 10D). Mice infected with the WT and PvIMC1g^TR^ parasites had similar survival curves (Fig. 10E). Additionally, the PvIMC1g^TR^ clone had comparable levels of gametocytemia, sex ratio, and exflagellation center formation to the WT parasites (Fig. 10F through H). The PvIMC1g^TR^ line also displayed a similar efficiency (76.5%) in ookinete conversion to the WT parasite (72.5%) during *in vitro* ookinete culture (Fig. 10I). Finally, the PvIMC1g^TR^ line showed similar competence to infect *An. stephensi* mosquitoes as the WT parasite in a direct mosquito feeding assay. Specifically, both the PvIMC1g^TR^ and WT parasite resulted in 100% infection prevalence in blood-fed mosquitoes (Fig. 10J). The oocyst density for the WT parasite (49.1/midgut) was also comparable with those for the comp (44.6/midgut) parasite (Fig. 10K). These findings demonstrate that PvIMC1g is functionally equivalent to PbIMC1g in the transgenic *P. berghei.*

## DISCUSSION

In this study, we have shown that PbIMC1g is a component of the IMC and interacts with the PHIL1 component IMC1c (21, 42). Deletion and mutagenesis analyses revealed that the proper IMC targeting of PbIMC1g depends on the IMCp domain, as described for other alveolins (29), while C-terminus and palmitoylation sites also contain information for effective IMC targeting. In addition, the core sub-repeat motif “EKI(V)V(I)EVP” is essential for PbIMC1g and IMC1c interaction. PbIMC1g KD impaired schizogony, inhibited male gametogenesis, and reduced ookinete gliding motility and infectivity similar to other alveolin mutants such as IMC1b and IMC1h (26, 27). In addition, we showed that PbIMC1g KD affects the localization and expression of the PHIL1 complex at the schizont and ookinete stages. Our studies prove that PbIMC1g is required for asexual stage schizogony and mosquito transmission.

Unlike some alveolins that are expressed in certain stages (e.g., IMC1a in sporozoites and IMC1b and IMC1i in ookinetes) (23, 26, 43–45), PbIMC1g is expressed in multiple but predominantly in proliferative and invasive stages. It is SPN-associated in merozoites and ookinetes. Furthermore, a previous study also observed the SPN association of PbIMC1g at the sporozoite stage (11). IMC proteins have been implicated as anchors for the glideosome components used for gliding motility and host cell invasion (7, 8). Previous reports showed that IMC1g co-purified with PHIL1 complex and GAP50 in IMC (16). Y2H analysis validated the interaction between PbIMC1g and a major PHIL1 complex protein, IMC1c. GAP50 and GAPMs are transmembrane proteins in the IMC (10, 18). Although we did not detect direct interaction between PbIMC1g and GAPM1-3 using conventional Y2H, GAPM1-3 are multiple transmembrane proteins, and their hydrophobic nature makes a split-ubiquitin membrane-based yeast two-hybrid system more suitable to test the protein interaction (46). The GAP50 is thought to recruit pre-complexed GAP45-MTIP-MyoA onto the outer membrane of the nascent IMC, while GAPMs of PHIL1 complex spanning both the outer and inner side of the IMC may serve as an anchor for fixing the actin-myosin motor to the SPN (10, 47–50). Therefore, the actin-myosin motor is recruited to the IMC by GAP50 to form the glideosome and is firmly fixed to the SPN by interacting with the IMC1g-PHIL1 complex.

Several mechanisms underlying IMC targeting have been proposed, including vesicle-mediated transport of proteins containing a signal peptide and six transmembrane domains (GAPM1-3), alveolin repeat-mediated IMC localization, coiled-coil (CC) domain-mediated IMC tethering, and palmitoylation-regulated IMC targeting (33, 47, 49, 51–53). We found that the IMCp domain harboring the repeat motif “EKI(V)V(I)EVP” was critical for the proper localization of the PbIMC1g protein. Although PbIMC1g does not possess a CC domain that is abundantly present in IMC proteins to facilitate protein-protein interactions (54, 55), the C-terminus of PbIMC1g is also required for efficient IMC targeting.

Protein palmitoylation, a reversible lipid modification, regulates protein stability, activity, and cell membrane binding and maintains cell shape in malaria parasites (25, 56, 57). Previous studies have shown that several IMC proteins are anchored to the membrane via palmitoylation, including the small heat shock protein 20 (HSP20), myosin light chain 1 (MLC1), MyoA, GAP45, PhIL1, inner membrane sub-compartment proteins, and IMC32 (34, 54, 58–64). However, this does not apply to all alveolins, such as PbIMC1c, which palmitoylation abolishing does not affect IMC/SPN targeting (65). The palmitomes of *P. falciparum* and *P. yoelii* both identified IMC1g as a palmitoylated protein (34, 66). This study confirmed that PbIMC1g and also PvIMC1g in the transgenic *P. berghei* were palmitoylated, and inhibition of protein palmitoylation with the broad-spectrum inhibitor 2-BP resulted in significantly reduced IMC targeting in schizonts. However, the mutagenesis assay revealed that the removal of N- or C-terminal palmitoylation sites reduced IMC/SPN targeting of PbIMC1g in schizonts but not in ookinetes. These results suggest that it is not the palmitoylation *per se* that is involved in IMC/SPN targeting, and the effect of reduced palmitoylation on IMC/SPN targeting in schizonts may be indirect. Several studies also found that palmitoylation regulates parasite life cycle progression (67–69). The DHHC protein family catalyzes protein palmitoylation in malaria parasites (70). Depletion of DHHC2, a major resident palmitoyl acyltransferase of the IMC, led to defective schizont segmentation and growth arrest, and IMC1g has been identified as a substrate of DHHC2 in the schizont stage (34). However, by using an edited Nmut strain, we found that mutations of the N-terminal palmitoylation sites of PbIMC1g had no effect on asexual stage growth but significantly reduced the formation of gametocytes. Interestingly, in this study, we found that PbIMC1g KD did not affect gametocytogenesis. Therefore, it is tempting to speculate that the wild-type IMC1g may not involved in gametocytogenesis and that perhaps mis-localized IMC1g in the Nmut strain somehow triggers a loss of gametocyte formation. Our results suggest that these palmitoylation sites of PbIMC1g could be exploited as a transmission-blocking target. Of the 12 DHHC protein family members, DHHC9 has been implicated in playing a role in gametocytogenesis (71). Whether PbIMC1g is a specific substrate for DHHC9 at this stage and the role of PbIMC1g palmitoylation in regulating gametocytogenesis will be valuable for future analysis. Overall, our study suggests that protein palmitoylation participates in IMC/SPN targeting of PbIMC1g protein, and the dysregulation of protein palmitoylation in PbIMC1g adversely affects sexual stage development.

Genome-wide functional screens in *P. berghei* and *P. falciparum*, as well as functional analysis of the PfIMC1g, all indicate that PbIMC1g is important or essential for asexual growth of the parasites (19, 72, 73). We have thus employed a KD system to examine the functions of the PbIMC1g protein. The IMC serves as the scaffold for forming and enveloping the nascent daughter cells during the replication process in apicomplexan parasites, such as *Toxoplasma gondii* and *Plasmodium* species (74, 75). Parasites deficient in IMC proteins, such as IMC32 in *T. gondii* and the basal complex component coordinator of nascent cell detachment (CINCH) in *P. falciparum*, exhibited impaired segmentation of daughter cells (54, 71, 76). Depletion of PfIMC1g showed minor defects in segmentation in *P. falciparum* (19). Here, we also found that PbIMC1g KD affected the schizogony in schizonts and IMC organization around the daughter merozoites, with significantly fewer merozoites, highlighting the importance of PbIMC1g in maintaining parasite cytoskeleton integrity. However, unlike the observation in *P. falciparum*, in which PfIMC1g depletion led to parasite death shortly after merozoite invasion (19), we found that PbIMC1g KD parasites could develop from the ring to schizont stage in *P. berghei*. Although this discrepancy may imply slight differences in IMC1g function in different parasite species, it is also possibly due to different levels of protein KD achieved in these parasites. Thus, a more robust KD or KO system will be valuable for testing the function of PbIMC1g in future work. Additionally, we found that PvIMC1g could completely complement PbIMC1g in the transgenic *P. berghei*, confirming the functional conservation of IMC1g among *Plasmodium* spp.

Male gametogenesis is a proliferative process in the mosquito vector essential for parasite transmission. PbIMC1g KD reduced the proportion of exflagellating male gametocytes, probably resulting from the defects in flagellar formation. Although, as a cytoskeleton protein, the influence on transcriptome is indirect, our global transcriptomic analysis revealed significant downregulation of microtubule (MT) cytoskeleton proteins involved in spindle formation, axoneme assembly, and male gamete formation (77–82). Interestingly, the mRNA levels of *pbimc1g* and PHIL1 complex genes (*pic5*, *phil1*, *gamp1*) were high in PbIMC1g KD gametocytes, which may reflect compensatory feedback in the same pathway. However, probably due to the knockdown system used in our study, the reduction of PbIMC1g KD on exflagellation is less than 20%. Thus, a robust knockdown system or a sexual stage-specific knockout strategy, such as promotor swapping, will be valuable for future studies to evaluate the role of PbIMC1g in male gametogenesis. Overall, our results showed the importance of PbIMC1g in male gametogenesis.

**Fig 11:**
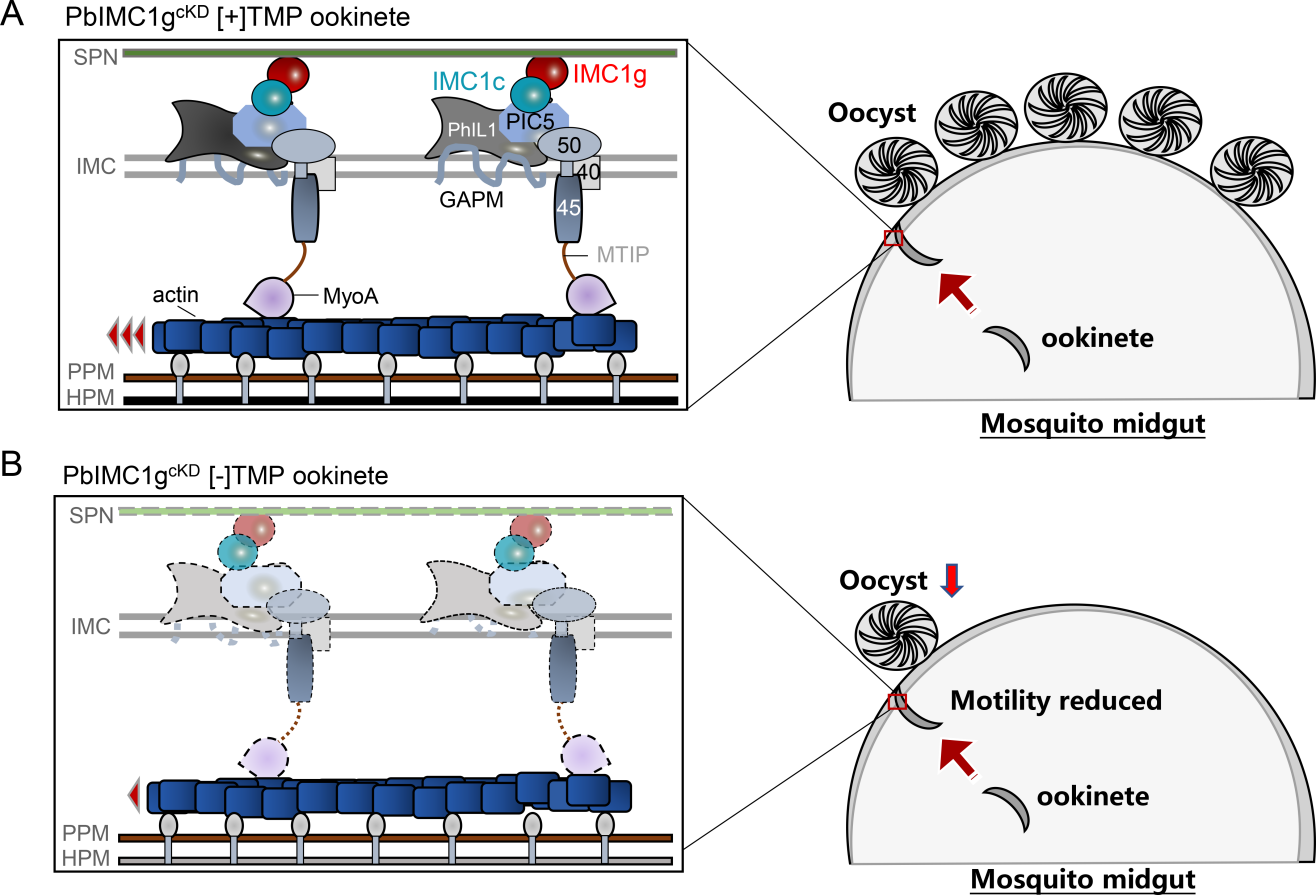
Diagram of the hypothesized mechanism of PbIMC1g in governing ookinete motility. (**A**) PbIMC1g under normally expressed condition (PbIMC1g^cKD^ [+] TMP). PbIMC1g anchors the glideosome to the SPN by interacting with PHIL1 complex. The motor complex provides the needed power for ookinete’s gliding and mosquito midgut invasion. (**B**) PbIMC1g under KD condition (PbIMC1g^cKD^ [−] TMP). PbIMC1g KD consequently reduces the stability of PHIL1 complex in IMC, which decreases the strength of the motor complex’s anchoring to the SPN, leading to reduced gliding motility of the ookinete.

Ookinetes undergo considerable constrictions as they migrate through the intestinal microbiota, peritrophic matrix, and the midgut epithelial cells of the mosquito (83, 84). It has been reported that the alveolins, such as IMC1a, -1b, and -1h, provide tensile strength to *P. berghei* ookinetes and sporozoites (23, 26, 27). Therefore, structurally weakened ookinetes, such as IMC1h-deficient ookinetes, were less able to invade successfully and more prone to damage during invasion (29). We observed a 40% of PbIMC1g KD parasites arrested at zygote and retort stages, highlighting the importance of IMC organization for ookinete morphology. Although we did not observe disorganized subpellicular MTs in PbIMC1g KD parasites using TEM, the downregulated expression of kinesins that function in MT dynamics (85) may account for the defects in ookinete conversion. Furthermore, the oocyst intensity was significantly reduced by over 50%, a reduction much more significant than that observed for the ookinete conversion, suggesting PbIMC1g may also participate in ookinete infectivity. To power the forward movement, the actin-myosin motor pulls the actin filaments and their attached adhesions in opposite ways (41, 86, 87), which requires the actin-myosin motor to be firmly fixed to the parasite cytoskeleton. Previous reports showed that IMC1 proteins contribute directly to the gliding motility of ookinetes and/or sporozoites, most likely by interaction with the glideosome (27). We have demonstrated that PbIMC1g interacted with the PHIL1 complex through IMC1c and might assist in anchoring the glideosome to the SPN (Fig. 11). We found that PbIMC1g KD dramatically reduced the expression of PHIL1 complex and glideosome components at the ookinete stage, probably responsible for the impaired directional motility and midgut invasion activity of the ookinetes.

In conclusion, we have defined the role of a conserved *Plasmodium* protein, IMC1g, with no previously known function in *P. berghei*. We discovered that IMC1g is present in all stages of *P. berghei*, predominantly expressed in schizonts. We showed that PbIMC1g is located in the IMC and interacts with the IMC1c of the PHIL1 complex. We found that the IMCp domain of PbIMC1g is required for proper IMC targeting, and the C-terminal and protein palmitoylation enhance the IMC targeting efficiency. We noted that PbIMC1g KD impacts schizogony, male gametogenesis, and ookinete development. In addition, PbIMC1g KD also affects ookinete motility and invasion. Finally, although PbIMC1g does not directly interact with any glideosome components, we found that PbIMC1g KD reduces the protein stability of IMC1c and GAPM1-3, which functions as a linker in stably docking glideosome to SPN. Therefore, PbIMC1g KD consequently affects the glideosome stability in SPN and reduces the gliding motility of ookinetes.

## MATERIALS AND METHODS

### Sequence analysis

The SMART software (http://smart.embl-heidelberg.de/) was used to analyze the conserved domains of PbIMC1g (PlasmoDB ID: PBANKA_1240600). To search for orthologs of *Plasmodium* IMC1g sequences in alveolates, annotated protein sequences were downloaded from plasmodb.org and https://www.ncbi.nlm.nih.gov for the following organisms: *P. falciparum*, *P. vivax*, *P. berghei*, *P. chabaudi*, *P. yoelii*, *P. knowlesi*, *T. gondii*, *Eimeria brunetti*, and *Babesia microti.* Amino acid sequence alignment was performed using MEGAX (88). The sequence similarities of PbIMC1g orthologs in apicomplexan parasites were performed using ENDscript 2.0 software (89). The phylogenetic analyses of PbIMC1g orthologs were performed using MEGA X and Eolview v3 software (90, 91). The residues of palmitoylation in IMC1g were predicted using online software CSS-Palm (csspalm.biocuckoo.org) (92).

### Parasite and mosquito maintenance

*P. berghei* ANKA 2.34 strain and the derived transgenic lines (PbIMC1g tagging line, PbIMC1g^HA^; PbIMC1g conditional KD line, PbIMC1g^cKD^; PbIMC1g N-terminal palmitoylation site mutant line, Nmut; and the PvIMC1g-expressing transgenic line, PvIMC1g^TR^) were maintained in 6- to 8-week-old Kunming outbred mice (Beijing Animal Institute, China) using standard protocols (93). The 6- to 8-week-old female BALB/c mice were used for all the phenotype analyses. The parasitemia of infected mice was monitored by microscopy of methanol-fixed and Giemsa-stained thin blood smears (94). Phenylhydrazinium chloride (PHZ; 80 µg/g mouse body weight, Sigma, MA, USA) was used to treat mice intraperitoneally (i.p.) 3 days before infection to induce hyper-reticulosis. Female *An. stephensi* Hor strain mosquitoes were reared at 25°C and 75% relative humidity with a 12-h light/dark cycle on 10% (wt/vol) glucose solution under standard laboratory conditions (93). For mosquito transmission experiments, blood feeding of mosquitoes was performed on anesthetized BALB/c mice at 3 dpi.

### Plasmid construction

The pYCm plasmid based on the CRISPR/Cas9 system was used for genomic editing of the *P. berghei* ANKA parasite. To generate the pYCm-PbIMC1g-HA (PbIMC1g protein C-terminal tagging) and pYCm-PbIMC1g-HA-DDD (PbIMC1g conditional knockout) vectors for tagging *pbimc1g* gene with *3×ha* or *3×ha-*ddd tags, respectively, we first amplified the C-terminal region (60 to 891 bp) of the coding region as the left arm and 1 to 778 bp from the 3′UTR region following the translation stop codon as the right arm using the primers listed in Table S2. The left and right arms were fused by overlapping PCR, with primers bearing the NcoI/SacII sites and 20 bp overlapping sequences necessary for the In-Fusion cloning system (Clontech, CA, USA). They were introduced using HindIII and AflII restriction sites into the pYCm vector. The DNA fragment encoding the 3×HA or 3×HA-DDD tags was inserted into NcoI/SacII sites between the left and right arms in-frame with the gene of interest, respectively. The sgRNA was designed to target the site close to the C-terminal coding region of the *pbimc1g* gene using the online program EuPaGDT (http://grna.ctegd.uga.edu/). Oligonucleotides for guide RNAs (sgRNAs) were mixed in pairs, denatured at 95°C for 3 min, annealed at room temperature for 5 min, and ligated into the *Bsm*BI site of the bypass plasmid to generate the final plasmids, pYCm-PbIMC1g-HA and pYCm-PbIMC1g-HA-DDD.

To generate the pYCm-PbIMC1g-Nmut plasmid for mutating the endogenous predicted N-terminal palmitoylation sites (cysteine 2, 10, and 11) to alanine, we amplified the homologous recombination region (−901 to 661 bp) containing the alanine mutations by overlapping PCR using primers listed in the Table S2 and ligated into the HindIII/AflII of the pYCm plasmid to generate the bypass plasmid. The specific sgRNA that targets the site close to the N-terminal coding region of the *pbimc1g* gene was designed and ligated into the bypass plasmid as described above to generate the final plasmid, pYCm-PbIMC1g-Nmut.

To create the pYCm-PvIMC1g-2×Myc plasmid for replacement of endogenous *pbimc1g* coding sequence with *pvimc1g* (PlasmoDB ID: PVX_079955) coding region, we first PCR amplified −901 to 25 bp of the 5′UTR region as left arm and 29 to 661 bp of the 3′UTR region as right arm using the primers listed in the Table S2. The overlapping PCR was performed using PCR products from 5′ and 3′ recombination regions to generate the final homologous region that bore the NcoI and SacII sites and ligated into the HindIII/AflII of the pYCm plasmid to generate the bypass plasmid. DNA fragment encoding the entire coding region of *pvimc1g* was amplified with specific primers and ligated into restriction enzyme sites NcoI/SacII of the bypass plasmid. The specific sgRNA that targets the site close to the N-terminal coding region of the *pbimc1g* gene was designed and ligated into the bypass plasmid as described above to generate the final plasmid, pYCm-PvIMC1g-2×Myc.

The pLyn-FRB-mCherry-nmd3-BSD plasmid was used to generate PbIMC1g deletion and palmitoylation site mutation constructs (95). Briefly, the endogenous promoter of *pbimc1g* (−1999 to −1 bp) was amplified from *P. berghei* gDNA using primers PbIMC1gpro-BglII-F and PbIMC1gpro-XhoI-R and inserted with BglII/XhoI upstream of the coding sequence. The human DHFR coding sequence was amplified from pSLI-2×FKBP GFP plasmid (95) with primers pLyn-WR-BamHI-F/pLyn-WR-HindIII-R and ligated into BamHI/HindIII sites, resulting in pLyn-FRB-mCherry-WR plasmid. Then, the *P. berghei* genomic DNA (gDNA) was used as the template to amplify truncations from the *pbimc1g* coding region, using primers listed in the Table S2. Each insert was cloned into the XhoI and MluI sites of the pLyn-FRB-mCherry-WR plasmid.

For recombinant protein expression in *Escherichia coli*, the DNA sequences encoding amino acids M_138_-K_301_ for GAPM1 (PBANKA_1338900), L_229_-Y_367_ for GAPM2 (PBANKA_0523900), W_2_-A_283_ for GAPM3 (PBANKA_1035400), and M_1_-R_459_ for IMC1c (PBANKA_1202000) were amplified from *P. berghei* cDNA using primers listed in Table S2 and subcloned into pET32a expression vector.

For yeast two-hybrid assay, the full-length coding sequence of PbIMC1g, PbIMC1c, PhIL1 (PBANKA_0204600), PIC5 (PBANKA_1409200), and GAPM1-3 and truncated versions of PbIMC1g protein were amplified using primers listed in Table S2, and ligated into EcoRI and BamHI sites of pGBKT7 and/or pGADT7 plasmids, respectively.

All the PCR amplifications and plasmid ligation were done with KOD-plus neo enzyme (Toyobo, Osaka, Japan) and the In-Fusion HD Cloning kit (Clontech). For parasite transfection, all constructs were purified using a Maxiprep kit (Qiagen, Dusseldorf, Germany).

### Recombinant protein expression and generation of antisera

The constructed pET32a-IMC1c, GAPM1, GAPM2, and GAPM3 expression vectors were expressed in Rosetta (DE3) cells and induced with 1 mM isopropyl-D-1-thiogalactoside (IPTG) at 19°C for 18 h. The recombinant 6×His-tagged IMC1c, GAPM1, -2, and -3 proteins were purified by Ni^2+^-NTA affinity chromatography (Thermo Fisher, MA, USA). The purified recombinant proteins were analyzed on 10% SDS-PAGE and confirmed by western blot using anti-His tag mAb (Sigma). Protein-specific antisera were raised in mice following standard procedures (96). Briefly, female BALB/c mice (*n* = 10) were initially immunized by subcutaneous injection with 50 µg of rGAPM proteins emulsified in complete Freund’s adjuvant (Sigma), followed by two booster injections at 2-week intervals with 30 µg of protein in incomplete Freund’s adjuvant. The same immunization procedure was performed for the PBS immunization control. Sera were collected and pooled 10 days after the last immunization.

### Parasite transfection, cloning, and diagnostic PCR

All transgenic parasites were generated from the *P. berghei* ANKA strain, and rodent parasite transfections were performed as previously described (97). Briefly, matured schizonts were purified from overnight *in vitro* culture on a 55% Nycodenz gradient and collected by centrifugation at 500 × *g* for 30 min, resuspended in 100 µL Amaxa Basic Parasite Nucleofector solution (Lonza, Basel, Switzerland) and added to 15 µg plasmid DNA. Schizonts were electroporated using Nucleofector 2B (Lonza) under U-033 program (98). Transfected parasites were immediately intravenously injected into a naïve mouse and were either exposed to 0.07 mg/mL pyrimethamine (Sigma) alone or pyrimethamine and 1 mg/mL trimethoprim (TMP; Sigma) 1 day after infection. Blood samples from infected mice were collected from the mouse tail tip, and the parasite gDNA was isolated from the blood samples using DNeasy blood kits (Qiagen) after washing off hemoglobin. To confirm the successful integration of the homologous templates, target locus recombination was detected by diagnostic PCR primers listed in the Table S2. Positive clones with targeted modifications were obtained by limiting dilution and used for phenotype analysis.

### Culture and purification of *P. berghei* stages

The purification of schizont, gametocytes, activated gametocytes, and ookinetes was achieved using a protocol described previously (97). Briefly, for schizont purification, PbIMC1g^HA^ parasite-infected red blood cells were collected from Kunming mice on day 3 p.i., and the parasites were cultured at 37°C overnight in schizont culture medium (RPMI 1640 with 50  mg/L penicillin, 50  mg/L streptomycin, 20% [vol/vol] heat-inactivated fetal calf serum [FCS]). The culture was then fractionated on a 55% [vol/vol] Nycodenz cushion to collect schizont-stage parasites. For gametocyte purification, parasites were injected into mice that had been PHZ-treated 3 days before infection. Three days after infection, parasites were enriched by 20 mg/L sulfadiazine (Sigma) treatment in drinking water for 48 h to eliminate asexually replicating parasites, and gametocyte-infected cells were purified on a 48% (vol/vol) Nycodenz gradient (99). The purified gametocytes were incubated with the ookinete culture medium (RPMI 1640, 50  mg/L penicillin, 50  mg/L streptomycin, 20% [vol/vol] FCS, 6 U/ml heparin, pH 8.0) at 25°C for 15 min to achieve activated gametocytes. For ookinetes, infected blood was harvested, cultured in a complete ookinete culture medium at 19°C for 24 h, and separated by 62% Nycodenz (97). For chemical inhibitor treatment, 2-BP (Sigma) at a final concentration of 100 µM was added to the ookinete culture 1 h after fertilization.

### Indirect immunofluorescence assay

The location of PbIMC1g-HA/DDD and PvIMC1g-2Myc protein was analyzed by IFA as described (100). Briefly, parasites were fixed with 4% paraformaldehyde and 0.0075% glutaraldehyde (Sigma-Aldrich) in PBS for 30 min at room temperature. The parasites were then permeabilized with 0.1% (vol/vol) Triton X-100 and neutralized with 0.1 mg/mL of sodium borohydride. After blocking with 5% skim milk, parasites were incubated with primary antibodies at room temperature for 1 h. The primary antibodies were as follows: mouse anti-HA mAb (1:500, Abcam, Cambridge, UK) or mouse anti-Myc mAb (1:500, Abcam), and rabbit antisera against CDPK1 (1:500), α-tubulin II (1:500), G377 (1:500), PSOP25 (1:500), GAP45 (1:500), and GAP50 (1:500). The polyclonal antibodies against CDPK1, α-tubulin II, G377, PSOP25, GAP45, and GAP50 were made in our laboratory. The samples were washed three times in 1× PBS and then incubated with Alexa Fluor 488-conjugated goat anti-mouse IgG antibodies (1:500, Invitrogen, MA, USA) and Alexa Fluor 555-conjugated goat anti-rabbit IgG antibodies (1:500, Invitrogen) as secondary antibodies, mounted using ProLong Diamond Antifade Mountant with DAPI (Thermo Fisher) and a coverslip, and then sealed with nail polish. All images were captured and processed on a Leica STELLARIS 5 fluorescence confocal laser scanning microscope (Leica, Wetzlar, Germany).

### Western blot analysis

Parasite protein pellets were collected from parental parasites, PbIMC1g^HA^ and PbIMC1g^cKD^. PbIMC1g-HA-DDD expression was achieved by adding 1 µM TMP to the culture medium or 1 mg/mL TMP supplied to the drinking water of mice. Proteins were extracted using 0.2% saponin in phosphate-buffered saline (PBS) with protease inhibitors and washed with PBS. After ultrasonication, the samples were centrifuged at 13,000 *g* for 5 min at 4°C, and the supernatants were run on a Novex WedgeWell 4%–12% gel (Thermo Fisher). The gel was then transferred to a PVDF membrane, blocked in Pierce Fast Blocking Buffer (Thermo Fisher), incubated with the primary antibody (1:3,000 anti-HA) in PBS with 3% bovine serum albumin (3% BSA/PBS), and then incubated with secondary antibodies diluted in Tris-buffered saline with Tween 20 (TBS-T). Proteins on the blot were visualized with the SuperSignal West Pico PLUS Chemiluminescent Substrate (Thermo Fisher) on Tanon 4200 (Tanon, Shanghai, China). The mouse anti-Hsp70 antibody, anti-GAPDH mAb, or anti-β-actin antibody was used for loading control. The protein band intensity was quantified using Fiji-Image J software (http://imagej.net/Fiji) (101).

The protein solubility analysis was performed as described (102). Briefly, the purified schizont/ookinete of the PbIMC1g^HA^ parasite (1 × 10^6^) was subjected to three rounds of freeze-thaw procedure (−80 to 24°C) in 200 µL of PBS containing 1% [vol/vol] protease inhibitor cocktail (Thermo Fisher). After centrifugation at 20,000 × *g* for 5 min at 4°C, the supernatant containing cytosolic soluble protein (FZ) was collected. The pellets were washed twice with ice-cold PBS, followed by further extraction in PBS containing 1% Triton X-100 for 30 min on ice. The Triton X-100-insoluble materials (membrane integral fraction) were washed twice with PBS, and proteins were further extracted by incubation with PBS containing 2% SDS (membrane interacting fractions) for 30 min at room temperature. The solubilized protein samples were subjected to immunoblot assays as described above.

### CuAAC click chemistry in schizonts and ookinetes

Freshly synchronized schizonts (42–45 hpi) or ookinetes were resuspended in schizont or ookinete culturing medium supplemented with 100 µM alkynyl palmitic acid (Alk-C16, TargetMol, MA, USA) and incubated at 37°C for 4 h to allow incorporation of Alk-C16. For the western blot assay, cells were washed three times with cold PBS, and the proteins were extracted by RIPA lysis buffer containing a complete protease inhibitor cocktail (Sigma). The labeled proteins were washed three times and resuspended with 1,000 µL of PBS, and copper-catalyzed alkyne azide cycloaddition (CuAAC) “click chemistry” reaction was performed in 30 µL of click chemistry reaction cocktail by sequentially adding 10 µL of biotin azide DMSO solution, 10 µL of copper (II) sulfate + protectant, and 10 µL of reducing agent. The reaction was allowed to take place for 90 min at room temperature. Then, the labeling reaction was added to 4 mL of methanol and 1 mL of chloroform, followed by 3 mL of water, and centrifuged for 10 min at 13,000–20,000 × *g* at 4°C. After removing the upper aqueous layer, 450 µL of cold methanol was added, followed by centrifugation for 5 min at 13,000–20,000 × *g* to pellet the proteins. The labeled samples were air-dried and resuspended in 800 µL of resuspend buffer (50 mM Tris, 150 mM NaCl, and 1% SDS), to which 100 µL of streptavidin agarose were added and incubated on a rotator for 2 h. The beads were washed two times with resuspension buffer, and the bound biotin-labeled proteins were eluted with 2× SDS buffer for western blot analysis.

IFA analysis was performed as described above to monitor the location profile of palmitoylated proteins in parasites at the schizont and ookinete stages. Briefly, the Alk-C16-labeled cells were fixed and permeabilized, as mentioned above. Fixed cells were washed three times with PBS, and CuAAC click reaction was performed in 100 µL of Click-iT reaction cocktail by sequentially adding 8 µL of biotin-azide (Thermo Fisher, final concentration 5 µM), 80 µL of 1× Click-iT reaction buffer, 2 µL of CuSO_4_ solution, and 10 µL of Click-iT buffer additive. The reaction was allowed for 30 min in the dark at room temperature. The cells were then stained with FITC-conjugated streptavidin polyclonal antibody (Thermo Fisher), mouse anti-HA mAb (Abcam), and rabbit anti-MSP1 (1:500) or rabbit anti-P25 (1:500), respectively. The samples were washed three times in 1× PBS and then incubated with Alexa Fluor 647-conjugated chicken anti-rabbit IgG antibodies (1:500, Invitrogen, MA, USA) and Alexa Fluor 555-conjugated goat anti-mouse IgG antibodies (1:500, Invitrogen) as secondary antibodies, mounted with ProLong Diamond Antifade Mountant with DAPI (Thermo Fisher), and analyzed using a Leica STELLARIS 5 fluorescence confocal laser scanning microscope (Leica).

### Yeast two-hybrid assay

Strain Y2Hgold (Clontech) was transformed with indicated pairs of pGBKT7 backbone bait plasmids and pGADT7 backbone prey plasmids by standard methods (TaKaRa, Dalian, China) (29). Strains were grown overnight in permissive (−Leu/−Trp) medium, normalized to OD_600_ = 2, and then spotted in three serial dilutions onto permissive (SD/−Leu/−Trp) and restrictive (SD/−Leu/−Trp/−His/−Ade) media. Growth was assessed after 3–5 days. Autoactivation was tested by co-transforming each pB27 fusion protein with an empty pP6 vector and co-transforming each pGBKT7 fusion protein with an empty pGADT7 vector and performing spot assays for each strain described above. The ability to grow without histidine and adenine requires the interaction of bait and prey hybrid proteins to drive transcription at two distinct GAL4-responsive loci. A negative control was performed using pGBKT7-Lam (which encodes the Gal4 BD fused with lamin) and pGADT7-T (which encodes the SV40 large T antigen).

### Real-time reverse transcript (RT)-PCR analysis

Total RNAs were isolated from purified PbIMC1g^cKD^ parasites (1 × 10^7^) with or without TMP treatment ([+]/[−] TMP) at schizont and ookinete stages using the QIAamp RNA Blood mini kit (Qiagen). cDNA was synthesized using the PrimeScript RT reagent kit after removing the contamination of gDNA using the DNA eraser (Thermo Fisher). Gene expression was quantified from 500 ng of total RNA using a SYBR Green fast master mix kit (TaKaRa). Analysis was conducted using a Bio-Rad iCycler iQ machine (Bio-Rad, CA, USA) with the following cycling conditions: 95°C for 5 min, followed by 40 cycles of 95°C for 15 s, and 60°C for 1 min. Three technical replicates and three biological replicates were performed for each assayed gene. The 18S ribosomal RNA (PBANKA_0521221) was used as a reference gene for real-time RT-PCR, and the fold change was calculated using the ΔΔCt method. The primers used for qPCR can be found in the Table S2.

### Parasite phenotype analysis

To determine the merozoite numbers per schizont, the PbIMC1g^cKD^ parasites were cultured *in vitro* as described in both [+]/[−] TMP conditions. After 15–16 h of culture, the mature schizonts were purified by 55% Nyocodenz, and the number of merozoites per schizonts was counted by Giemsa-stained thin smear.

To induce gametocyte formation, 5 × 10^6^ parasites of the PbIMC1g^cKD^ line were injected intraperitoneally (i.p.) into mice pre-treated with PHZ. The gametocytemia and the male/female ratio were estimated by microscopy using Giemsa-stained thin smears. For measuring microgametocyte exflagellation, 10 µL of blood from a tail vein was added to 40 µL of gametocyte activation medium (RPMI 1640 containing 25 mM HEPES, 4 mM sodium bicarbonate, 20% FCS, 100 µM xanthurenic acid, pH 7.8) and incubated for 15 min at 25°C. The number of exflagellation centers per 10 fields at 400× magnification was counted under a microscope. To stabilize the PbIMC1g-HA-DDD expression, 1 µM of TMP dissolved in DMSO was added to the gametocyte activation medium.

*In vitro* ookinete differentiation was performed as described previously (97). Briefly, 10 µL mouse blood with 8%–10% gametocytemia was collected from PbIMC1g^cKD^ parasite-infected mice via orbital sinus and cultured with 90 µL of ookinete culture medium (RPMI 1640, 25 mM HEPES, 20% FCS, 100 mM XA, pH 8.0) for 24 h either [+]/[−] 1 µM TMP supplement at 20°C. For ookinete conversion analysis, samples from 24 h of [+]/[−] 1 µM TMP culture were collected and incubated with the anti-Pbs21 monoclonal antibody, followed by fluorescently labeled secondary antibody for 1 h. The ookinete conversion rate was calculated as the number of mature ookinetes (stage V) over the total number of zygotes, retorts, and ookinetes.

To analyze mosquito transmission, 30–50 *An. stephensi* mosquitoes were allowed to feed for 1 h on anesthetized, infected mice with an asexual parasitemia of approximately 12% and a comparable number of gametocytes as determined on Giemsa-stained blood films. To assess midgut infection, the mosquitoes were allowed to feed on either 10% (wt/vol) glucose solution alone or 10% (wt/vol) glucose solution and 1 µM TMP. Approximately 50 mosquitoes were dissected on day 10 post-feeding, and oocysts were stained with 0.5% mercurochrome and counted by Olympus microscope using a 63× oil immersion objective.

### Ookinete Matrigel motility assays

Ookinete motility was assessed as previously described (103). All procedures were performed in a temperature-controlled room at 19°C. Briefly, ookinete cultures were added to an equal volume of Matrigel (BD, NJ, USA) on ice, mixed thoroughly, transferred onto a slide, covered with a coverslip, and sealed with nail polish. The slide was placed at 19°C for 30 min before observation under a microscope. After identifying a field containing ookinetes, time-lapse videos (100×; 1 frame every 20 s for 30 min) were taken to monitor ookinete movement on a Leica Microsystems GmbH STELLARIS 5 microscope fitted with a DMI8 automated digital camera controlled by Leica Application Suite X software (Leica). Time-lapse movies were analyzed using the Fiji software and the Manual Tracking plugin. Motility speed was calculated by dividing the distance an ookinete moved by the time takes. Results are representative of two independent ookinete cultures at least.

### Electron microscopy

Purified schizonts and ookinetes of PbIMC1g^cKD^ parasites cultured under [+]/[−] TMP conditions were adjusted to ~50% parasitemia with uninfected erythrocytes and fixed in 2.0% glutaraldehyde in 0.1 M phosphate buffer and processed as described (104). Briefly, the samples were post-fixed in osmium tetroxide, treated with uranyl acetate solution, washed with H_2_O, dehydrated in grades of alcohol, and embedded in LR White resin (Sigma). Thin sections were stained with uranyl acetate and lead citrate before being observed with a Hitachi HT7700 transmission electron microscope (Hitachi, Tokyo, Japan) at 120 kV.

### RNA-seq and data analysis

About 10^8^ purified activated gametocytes from PbIMC1g^cKD^ [+]/[−] TMP parasites were lysed in 10× volumes of TRIzol (Thermo Fisher) and used for total RNA extraction. The experiment was repeated at 3-week intervals to obtain samples as biological duplicates. Sample preparations were performed as described (105). Briefly, mRNA was enriched using poly-T oligo-attached magnetic beads. Reverse transcription was performed with SuperScript II (Invitrogen) using random primers. The reaction was then treated with RNaseH to remove the RNA template and cleaned using DNA Clean and Concentrator Kit (Zymo Research). The clustering of the index-coded samples was performed on a cBot Cluster Generation System using TruSeq PE Cluster Kit v3-cBot-HS (Illumina) according to the manufacturer’s instructions. After cluster generation, the library preparations were sequenced using the Illumina platform. RNA-seq raw data were initially filtered by fastq to obtain high-quality, clean data. Adapter trimming was performed using cutadapt version 1.13 (http://cutadapt.readthedocs.io/en/stable/guide.html). Processed reads were aligned to the *P. berghei* ANA genome (assembly PlasmoDB-67) with Hisat2 v2.0.4 (106) and deduplicated according to UMI using UMI-tools (v1.0.0) (107). Read counts were obtained with HTSeq v0.9.1. Differential expression analysis of genes between the PbIMC1g^cKD^ parasites cultured in both [+] and [−] TMP conditions was determined using the DESeq R package (1.18.0). The *P-*values were adjusted using the Benjamini-Hochberg procedure, which controls the false discovery rate. Genes with an adjusted *P* value < 0.05 found by DESeq were assigned as differentially expressed. GO enrichment analysis was implemented using the GOseq R package (108).

### Statistical analyses

All statistical analyses were performed using GraphPad Prism 9.0 (GraphPad Software). Two-tailed Student’s *t*-test or Mann–Whitney *U* test was used to compare differences between transgenic strains in the presence or absence of TMP or between the wild-type and the mutant strain. The *P*-value in each statistical analysis was indicated within the figures.

## Data Availability

The RNA-seq raw data have been deposited in the GEO database (accession number: GSE269994).

## SUPPLEMENTAL MATERIAL

Supplemental material mbio.02652-24-s0001.pdf Supplemental figures and captions for supplemental tables and movies.

Table S1 mbio.02652-24-s0002.xlsx Global transcriptional analysis of activated gametocytes from PbIMC1g^cKD^ [+]/[−] TMP parasites by RNA-seq.

Table S2 mbio.02652-24-s0003.xlsx Oligonucleotides used in the study.

Movie S1 mbio.02652-24-s0004.avi The gliding motility of PbIMC1g^cKD^ [+] TMP parasites.

Movie S2 mbio.02652-24-s0005.avi The gliding motility of PbIMC1g^cKD^ [−] TMP parasites.

ASM does not own the copyrights to Supplemental Material that may be linked to, or accessed through, an article. The authors have granted ASM a non-exclusive, world-wide license to publish the Supplemental Material files. Please contact the corresponding author directly for reuse.
