# Supplementary material for: An inner membrane complex protein IMC1g in Plasmodium berghei is involved in asexual stage schizogony and parasite transmission
Source: mBio. 2024 Nov 22;16(1):e02652-24. doi: 10.1128/mbio.02652-24 (PMC11708024; doi:10.1128/mbio.02652-24)
Supplement: Supplemental material — Supplemental figures and captions for supplemental tables and movies. [file mbio.02652-24-s0001.pdf]

SUPPLEMENTAL MATERIAL

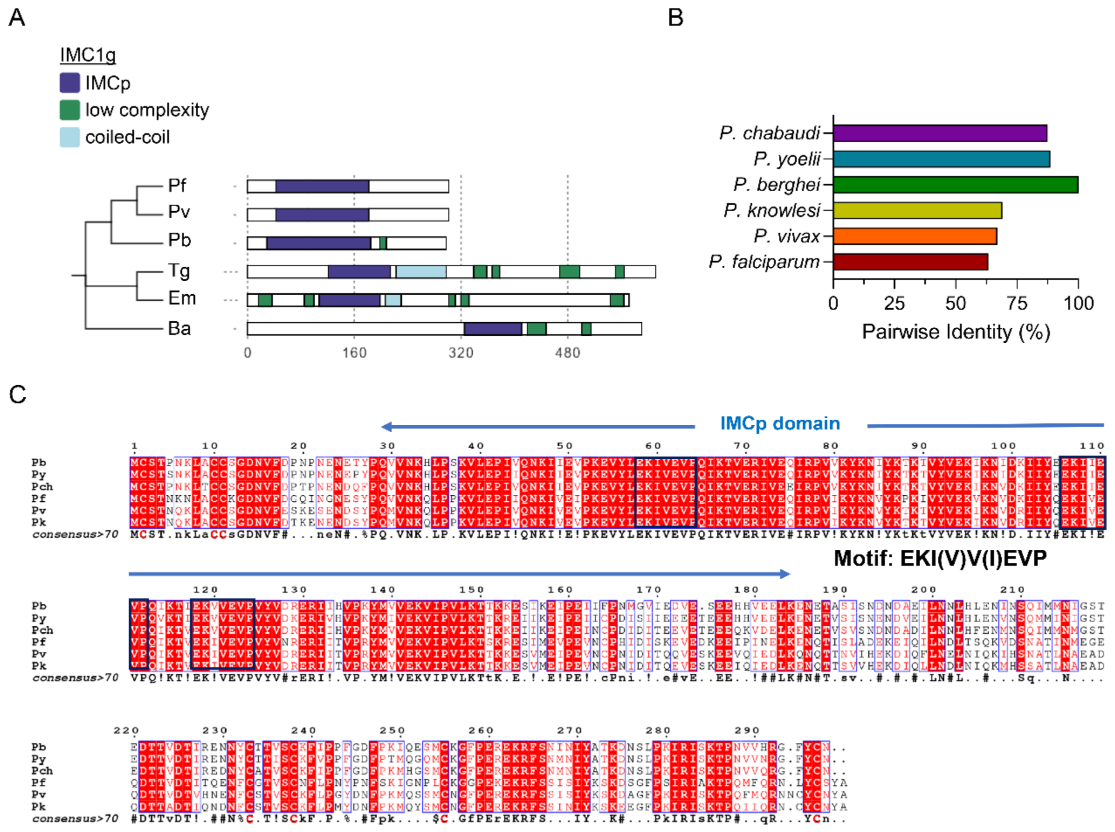

FIG S1. Sequence and structure of IMC1g. (**A**) Phylogenetic tree of PbIMC1g and blastp-searched proteins across species. The following sequence of IMC1g protein was used: *P. falciparum* 3D7 (Pf), PlasmoDB ID: PF3D7_0525800; *P. vivax* Sal-I (Pv), PVX_079955; *P. berghei* ANKA (Pb), PBANKA_1240600; *Toxoplasma gondii* ME49 (Tg), GenBank no. XP_002368044.1; *Eimeria brunetti* (Em), CDJ53566.1; *Babesia microti* strain RI (Ba), XP_012648570.1. (**B**) Multiple pairwise alignments of the amino acid sequence of IMC1g orthologues in *Plasmodium* spp. The sequence of *P. chabaudi chabaudi* (Pch, PCHAS_1241000), *P. yoelii* 17X (Py, PY17X_1243800), *P. knowlesi* strain H (Pk, PKNH_1007000) were obtained from the PlasmoDB website. (**C**) Multiple sequence alignment of IMC1g protein sequences from Pb, Py, Pch, Pf, Pv, and Pk using MUSCLE. The blue arrows marked the predicted IMCp domain of IMC1g protein. The sub-repeat 'EKI(V)V(I)EVP' motif within the IMCp domain is with black boxes.

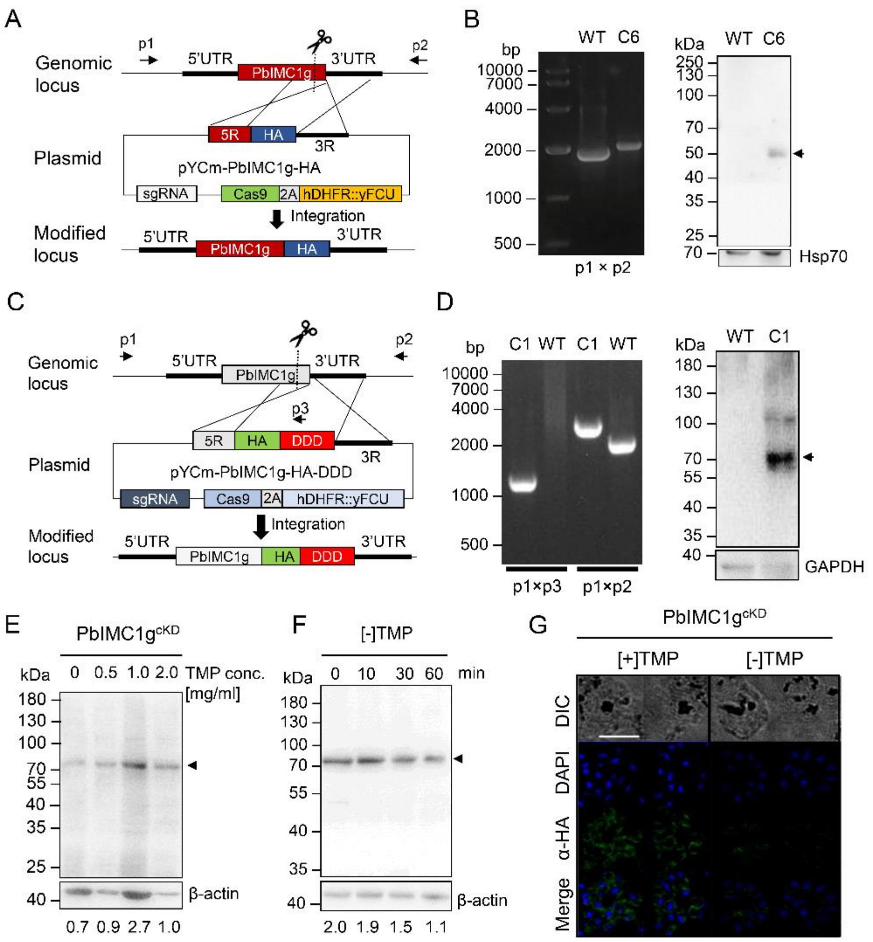

FIG S2. Generation of PbIMC1g^{HA} and PbIMC1g^{cKD} transgenic parasite line. (**A, C**) The schematic shows the strategy for generating PbIMC1g^{HA} (**A**) and PbIMC1g^{cKD} (**C**) parasites. 3×HA, triple hemagglutinin tag. DDD, a dihydrofolate reductase (DHFR)-based destabilizing domain. The position of the primers used in diagnostic PCR is indicated with arrows. (**B, D**) Validation of generated transgenic PbIMC1g^{HA} (**B**) and PbIMC1g^{cKD} (**D**) parasite line. Confirmatory PCR of unmodified wild-type (WT) and transgenic knock-in (KI) cell line PbIMC1g^{HA} clone 6 (C6) or PbIMC1g^{cKD} line clone 1 (C1) to check integration at the 3' locus (left panel). The expression of PbIMC1g-HA or PbIMC1g-DDD protein was confirmed by western blot using anti-HA mAb (right panel). The arrows indicate PbIMC1g-HA (37.8 kDa) and PbIMC1g-DDD (59.4 kDa) protein. Hsp70 or GAPDH was used as a loading control. (**E**) Western blot analysis of PbIMC1g^{cKD} transfectants shows that PbIMC1g levels decrease in a TMP dose-dependent manner. β actin was used as a loading control. The arrowhead indicates PbIMC1g-DDD protein. The relative signal intensity ratios of PbIMC1g-DDD/β-actin calculated with the Image J software are shown below the gel image. (**F**) Western blot analysis of PbIMC1g^{cKD} transfectants shows that PbIMC1g levels decrease after the removal of TMP. β-actin was used as a loading control. The arrowhead indicates PbIMC1g-DDD protein. The relative signal intensity ratios of PbIMC1g-DDD/β-actin calculated with the Image J software are shown below the gel image. (**G**) Fluorescent microscopy of PbIMC1g^{cKD} parasites cultured in the presence (left) or absence (right) of TMP (1 μM). Scale bar, 5 μm.

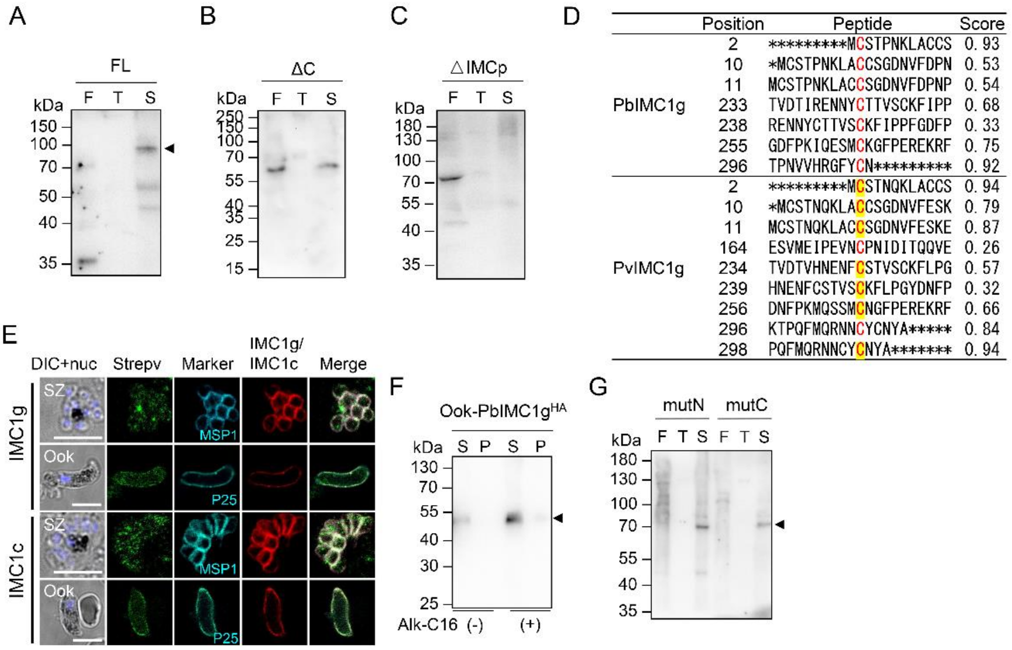

FIG S3. Solubility assay of PbIMC1g recombinant proteins. Solubility assay detecting membrane association of FL (**A**), ΔC (**B**), ΔIMCp (**C**), and mutN and mutC (**G**) using different detergents. Cytosolic soluble proteins are in Freeze-thaw (F), integral membrane proteins in Triton X-100 buffer (T), and peripheral membrane proteins in 2% SDS buffer (S). (**D**) CSS-Palm software predicted Palmitoylation sites in PbIMC1g and PvIMC1g proteins. The putative palmitoylated sites are labeled with red letters, and the position-conserved sites in PvIMC1g are further marked with yellow colors. (**E**) Click chemistry method detecting palmitoylation of PbIMC1g in PbIMC1g^{HA} parasites in the schizont (SZ) and ookinete (Ook) stages. The alkynyl palmitic acid (Alk-C16) labeled PbIMC1g-HA or PbIMC1c proteins were stained with Alexa Fluor 488 conjugated streptavidin (Strepv, green), parasite plasma membrane (PPM) marker MSP1 (cyan) or P25 (cyan), and anti-HA mAb (IMC1g, red) or anti-PbIMC1c sera (IMC1c, red). Merged images for Strepv, PPM marker, and IMC1g or IMC1c are shown in the right column. Scale bar, 5 µm. (**F**) Click chemistry method detecting palmitoylation of PbIMC1g-HA protein in PbIMC1g^{HA} parasites at ookinete (Ook) stages. The captured palmitoylated proteins were analyzed by western blotting using anti-HA mAb. S, supernatant; P, pellet. The arrowhead indicates PbIMC1g-HA protein.

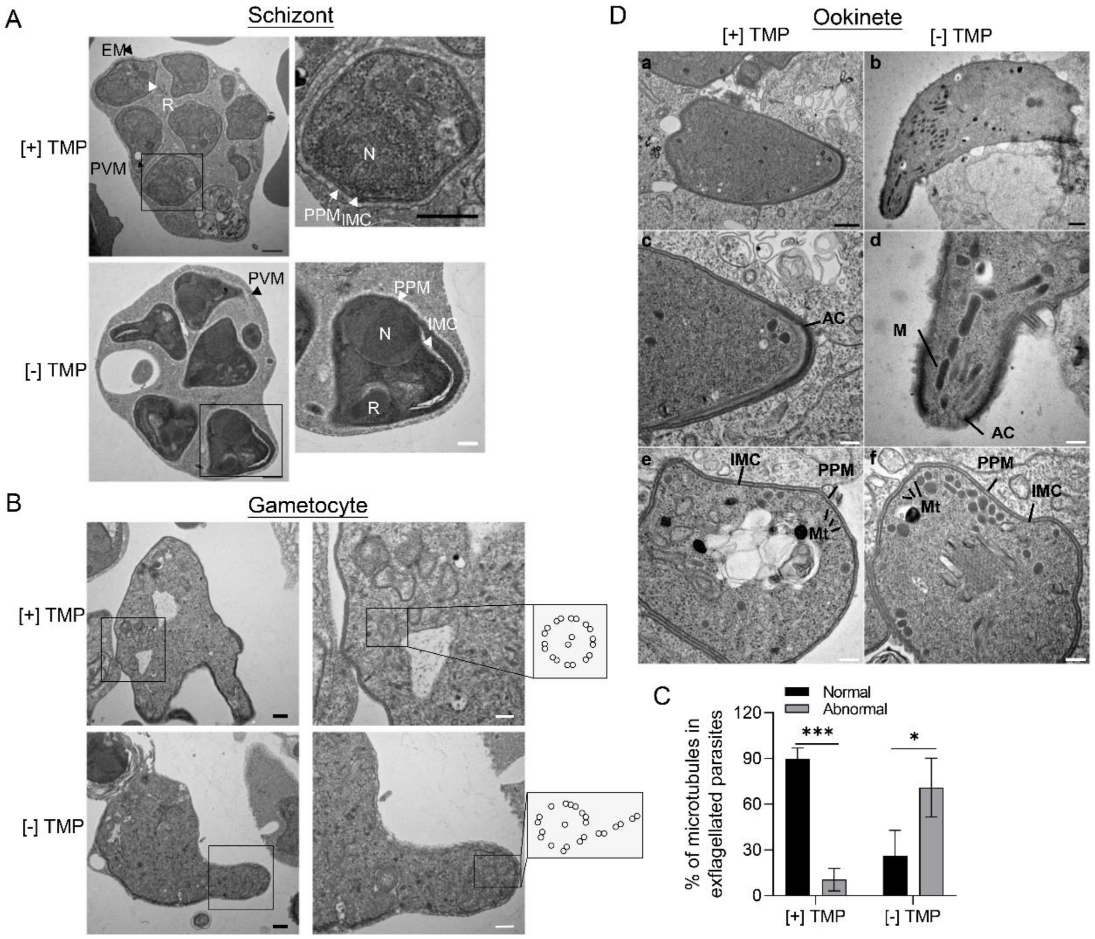

FIG S4. TEM images of schizont, activated gametocyte, and ookinete stage of PbIMC1g^{cKD} [+] /[-] TMP parasites. (**A**) TEM images of matured schizont stage PbIMC1g^{cKD} [+] /[-] TMP parasites from in *vitro* schizont cultures. In both images, the different membranes are indicated as follows: erythrocyte (EM, black arrowhead), parasite vacuole (PVM, black arrowhead), parasite plasma (PPM, white arrow), and inner membrane complex (IMC, white arrow). N, nucleus; R, rhoptry. Representative of 2 experiments. experiment 1, n=20; experiment 2, n=15. Scale bars, 500 nm (black), 200 nm (white). (**B**) TEM images of male gametogenesis of PbIMC1g^{cKD} [+] /[-] TMP parasites at 8 mpa. Scale bars, 500 nm (black), 200 nm (white). Schematics of axonemes of PbIMC1g^{cKD} [+] /[-] TMP parasites at 8 mpa were shown in the right panel. (**C**) Quantifications of exflagellated PbIMC1g^{cKD} [+] /[-] TMP parasites with normal or abnormal microtubules. (**D**) TEM images of PbIMC1g^{cKD} [+] TMP (a, c, e) and [-] TMP (b, d, f) parasites at the ookinete stage. **a-d.** Longitudinal section of a crescent-shaped [+]/[-] TMP ookinete. AC, apical complex; M, micronemes. **e-f.** Cross section through the periphery of the anterior complex of a [+] TMP (e) and a [-] TMP (f) parasite showing similar substructure consisting of the outer parasite plasma membrane (PPM) and the underlying inner complex membrane (IMC). Mt, subpellicular microtubules. Scale bars: 500 nm (black), 200 nm (white).

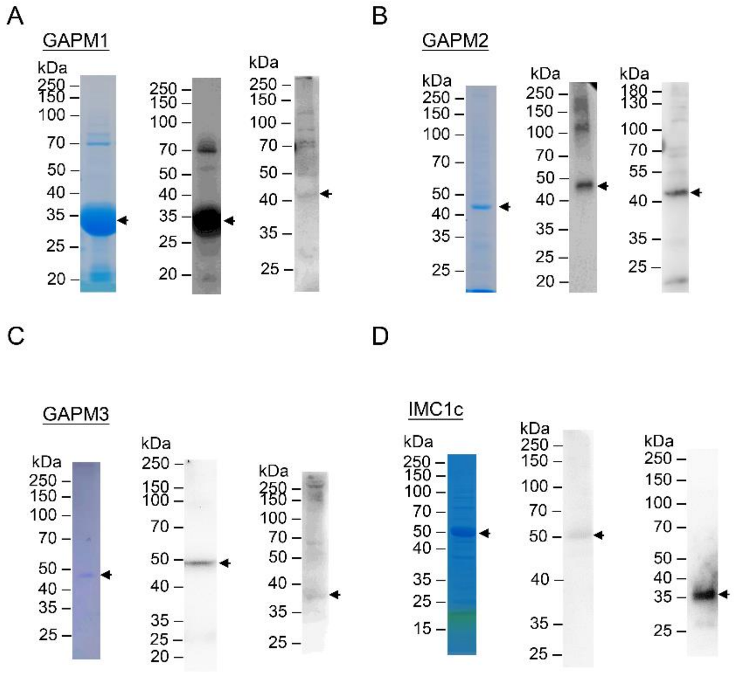

FIG S5. Purification and detection of the PHIL1 complex recombinant and endogenous proteins. (**A-D**, left panel) Purified recombinant GAPM1 (**A**), GAPM2 (**B**), GAPM3 (**C**), and IMC1c (**D**) proteins were separated on 10% SDS-PAGE and stained with Coomassie brilliant blue. (**A-D**, middle panel) Western blot analysis of PHIL1 recombinant proteins (middle panel). The blots were probed with the anti-his tag monoclonal antibody. (**A-D**, right panel) Western blot analysis of protein extracts from mixed blood stage. The blots were probed with the anti-GAPM1 (**A**), GAPM2 (**B**), GAPM3 (**C**), and IMC1c (**D**) sera, respectively. Arrows indicate recombinant and endogenous PHIL1 components. rGAPM1-his, 37.7 kDa; rGAPM2-his, 35.3 kDa; rGAPM3-his, 51.7 kDa; rIMC1c-his, 51.5 kDa; GAPM1, 33.7 kDa; GAPM2, 42 kDa; GAPM3, 32.7 kDa; IMC1c, 32.6 kDa.

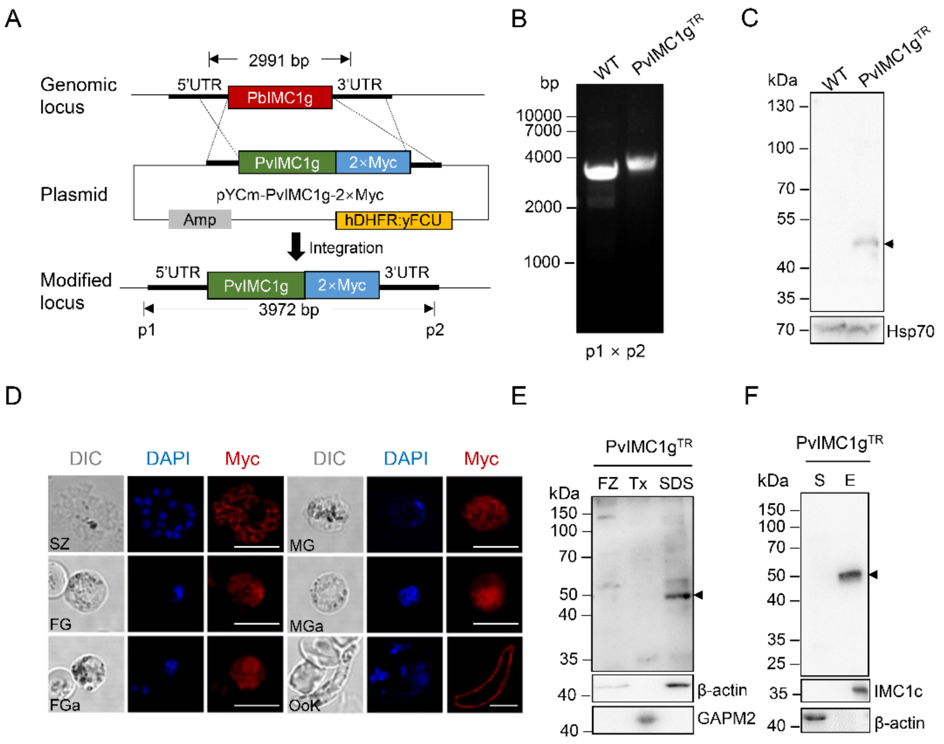

FIG S6. Generation of PvIMC1g^{TR}-transgenic parasites. (**A**) Schematic showing the replacement of the *pbimc1g* coding region with *pvimc1g* in *P. berghei* and simultaneously tagging PvIMC1g with a 2×Myc tag. The primers used to verify the integration are indicated by arrows. Thick lines indicate 5' and 3' UTRs. (**B**) PCR confirmation of the *pvimc1g* coding sequence integration at the *pbimc1g* genomic locus. C10 is a clone of the PvIMC1g^{TR} transgenic parasite line. PCR products of WT and PvIMC1g^{TR} show the native (2991 bp) and modified region (3972 bp). (**C**) Western blot confirmation of PvIMC1g-2Myc protein expression in PvIMC1g^{TR} transgenic parasites using anti-Myc mAb. Hsp70 as loading control. The arrow indicates PvIMC1g-2Myc protein, ~38.5 kDa. (**D**) IFA of PvIMC1g^{TR} parasite in schizont (SZ), gametocyte (FG, female gametocyte; FGa, activated female gametocyte; MG, male gametocyte; MGa, activated male gametocyte), and ookinete (Ook) stages. Parasites were co-stained with anti-Myc antibody and DAPI. Scale bar, 5 μm. (**E**) Western blot analysis of Freeze-Thaw (FZ), 0.1% Triton X-100 detergent (Tx), and SDS fractionations. The PvIMC1g-2Myc band was visualized using mouse anti-Myc mAb. β-actin and GAPM2 were used as loading controls for SDS and Tx fractions, respectively. The arrowhead indicates recombinant PvIMC1g-2Myc protein. (**F**) Co-IP analysis of PvIMC1g-2Myc ookinete lysates. Ookinete lysates of PvIMC1g^{TR} parasite were precipitated with Pierce Magnetic c-Myc-Tag co-IP kit and followed by immunoblotting with anti-Myc mAb, anti-IMC1c sera, and anti-β actin antibody, respectively. S, supernatant; E, the precipitated elutes. The arrowhead indicates recombinant PvIMC1g-2Myc protein.
